# Phylogenomics and species delimitation of the economically important Black Basses (*Micropterus*)

**DOI:** 10.1038/s41598-022-11743-2

**Published:** 2022-06-06

**Authors:** Daemin Kim, Andrew T. Taylor, Thomas J. Near

**Affiliations:** 1 Department of Ecology and Evolutionary Biology, Yale University, P.O. Box 208106, New Haven, CT 06511 USA; 2 Department of Biology, University of Central Oklahoma, Edmond, OK 73034 USA; 3 Peabody Museum, Yale University, New Haven, CT 06511 USA; 4 Present Address: Department of Biology, University of North Georgia, Dahlonega, GA 30597 USA

**Keywords:** Phylogenetics, Population genetics, Speciation, Taxonomy, Ichthyology

## Abstract

Informed management and conservation efforts are vital to sustainable recreational fishing and biodiversity conservation. Because the taxonomic rank of species is important in conservation and management strategies, success of these efforts depends on accurate species delimitation. The Black Basses (*Micropterus*) are an iconic lineage of freshwater fishes that include some of the world’s most popular species for recreational fishing and world's most invasive species. Despite their popularity, previous studies to delimit species and lineages in *Micropterus* suffer from insufficient geographic coverage and uninformative molecular markers. Our phylogenomic analyses of ddRAD data result in the delimitation of 19 species of *Micropterus*, which includes 14 described species, the undescribed but well-known Altamaha, Bartram’s, and Choctaw basses, and two additional undescribed species currently classified as Smallmouth Bass (*M. dolomieu*). We provide a revised delimitation of species in the Largemouth Bass complex that necessitates a change in scientific nomenclature: *Micropterus salmoides* is retained for the Florida Bass and *Micropterus nigricans* is elevated from synonymy for the Largemouth Bass. The new understanding of diversity, distribution, and systematics of Black Basses will serve as important basis for the management and conservation of this charismatic and economically important clade of fishes.

## Introduction

Recreational fishing is globally among the most popular outdoor activities^1^ and has high socio-economic importance^2^. Approximately 47 billion fishes were landed recreationally in each year between the late 1990s to early 2000s worldwide^3^ and in the United States (USA) anglers spent $46.1 billion USD in 2016^4^. While recreational fishing is expected to increase globally^5^, threats to the biodiversity that comprise recreational fisheries include habitat degradation, overfishing, invasive species, and climate change^6–9^. Informed management and conservation efforts are vital to sustainable recreational fishing and biodiversity conservation. Given that species is the taxonomic rank overwhelmingly targeted in conservation and management strategies^10^, success of these efforts depends on accurate species delimitation^11^.

Contemporary genomics and analytical methods complement traditional morphology-based species delimitation of fishes (e.g., Ref.^12^). The application of genomics to species delimitation facilitates the resolution of genetic population structure and the discovery of distinct evolutionary lineages^13^. Many groups of vertebrates are the subjects of genomic-based species discovery, including charismatic megafauna such as finless porpoises^14^, giraffes^15^, and orangutans^16^. Phylogenomics and population genomics provide important scientific insight on economically important species that includes revealing substantial intra- and interspecific differentiation among whitefishes (*Coregonus*) in Lake Superior^17^, the discovery of multiple distinct populations of Atlantic Cod (*Gadus morhua*) in northwestern Europe that challenges the current designations of management units^18^, and a complex history of secondary contact and linked selection in Atlantic Salmon (*Salmo salar*) across Europe and North America^19^. However, there is a surprising lack of genomic-based investigations of species delimitation and range-wide intraspecific genomic diversity for freshwater fishes of high international recreational importance. Failure to incorporate accurate species delimitation and information on population structure in fisheries management increases the risk of extinctions by overlooking rare and undiscovered species^20^ and the potential to disturb native genetic diversity through the introduction of non-native species or individuals from genetically divergent populations^21^.

Black Basses (*Micropterus*) are a lineage of freshwater fishes endemic to North American river basins east of the Rocky Mountains that includes species which are some of the world’s most popular targets for recreational fishing. The three species complexes of *Micropterus* most popular with anglers*,* Largemouth Bass (currently *M. salmoides*), Smallmouth Bass (*M. dolomieu*), and Spotted Bass (*M. punctulatus*) emerged as quintessential sportfishes in the nineteenth century^22–24^. Because of their popularity, species of *Micropterus* have been stocked throughout the world since the early 1800s^23,25^. Early stockings were poorly documented, but occurred outside the native ranges of species as well as across river basins within the range of a species, often without regard for species diversity within *Micropterus*^23^. Economic benefits stemming from recreational fishing and aquaculture have spurred introductions of the Largemouth Bass into at least 57 countries across every continent except Antarctica^26^, resulting in this species being recognized as both one of the world’s most popular freshwater sport fishes and among the world's most invasive species^27^; Global Invasive Species Database www.iucngisd.org/gisd/.

Although all of the three species complexes of *Micropterus* most popular with anglers have expansive native geographic distributions, they are not immune to the effects of stocking and mixing of distinct lineages or populations, which risks the erosion of local adaptation, interruption of co-adapted gene complexes, and species extinction. Present day management practices continue to promote stocking of the Florida Bass (currently recognized as *Micropterus floridanus*) into the range of the closely related Largemouth Bass to promote increased growth rate or maximum sizes^28–31^. Similarly, distinct lineages and populations of the Smallmouth Bass have been recognized since 1940 with the discovery and description of the Neosho Bass (*M. dolomieu velox*) as a subspecies of Smallmouth Bass^32^. Recent population genetic assessments illustrate that stocking of the more widespread Smallmouth Bass (*M. dolomieu dolomieu*) into impoundments has led to genetically admixed populations within the narrow native range of the Neosho Bass^33,34^. Despite the fact that the Alabama Bass (*M. henshalli*) was recognized as a distinct species nearly 15 years ago^35^, it is still introduced outside of its native distribution where it hybridizes with other species of *Micropterus* with the threat of regional extirpation of native species of *Micropterus*^36,37^.

In this study, we investigate the phylogeny and delimitation of species of *Micropterus* using a double digest restriction-site associated DNA (ddRAD) genomic dataset sampled with comprehensive geographic coverage of all recognized species in the clade. We rely on an operational species concept that views separately evolving metapopulation lineages as the sole criterion for the recognition of species^38,39^. Our analyses not only provide a perspective on species diversity in *Micropterus* that differs from those accepted by both ichthyologists and fisheries scientists^24,40^, but also dramatically reveal that the scientific names *Micropterus salmoides*^41^ and *Micropterus floridanus*^42^ have been incorrectly applied to the Largemouth Bass and Florida Bass over the past 75 years^43^. In addition to a new delimitation of Black Bass species, the genomic analyses provide a reconstruction of their evolutionary history and an important basis for the management and conservation of this economically and culturally important recreational fishery resource.

## Materials and methods

### Specimen sampling and ddRAD data collection and assembly

**Figure 1:**
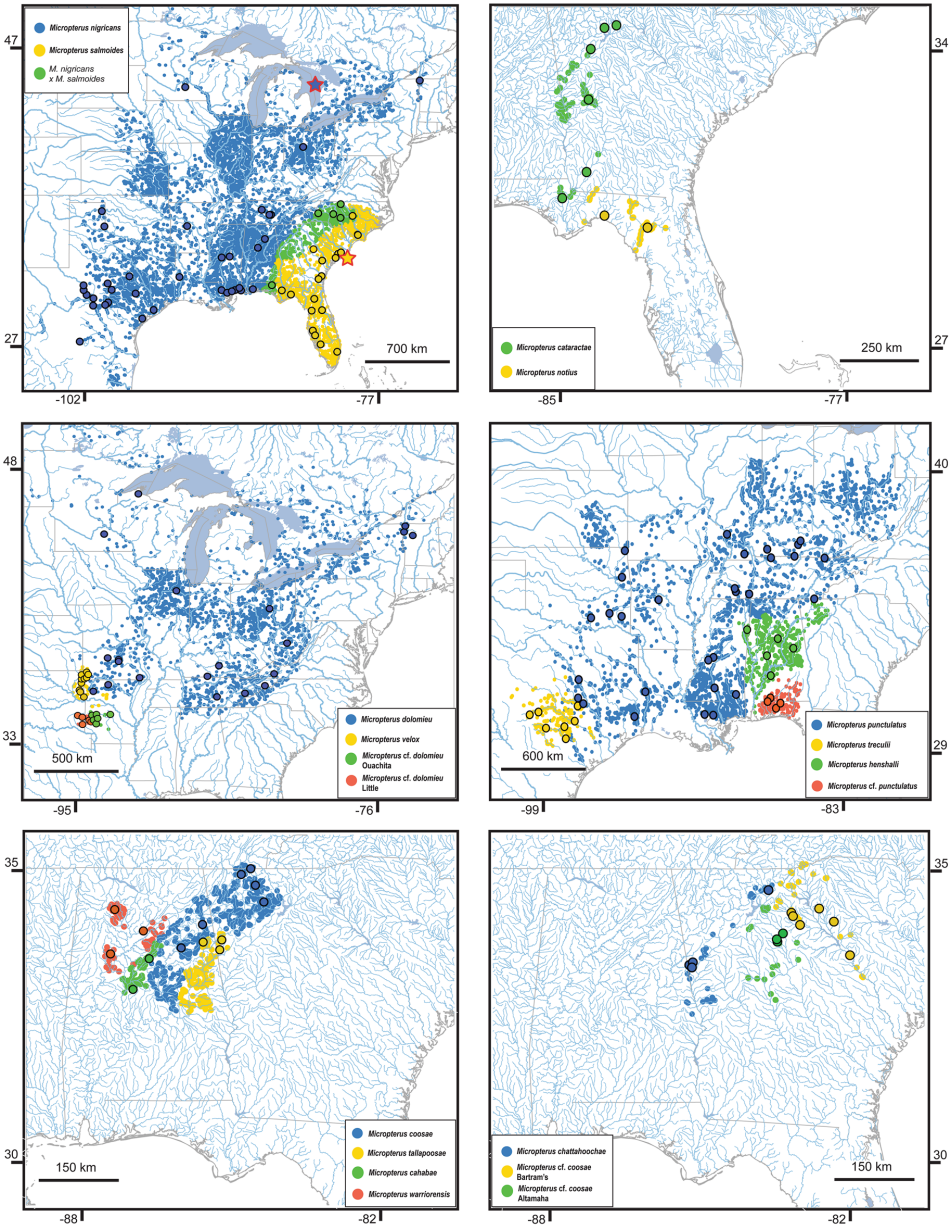
Geographic ranges of all species in *Micropterus* as delimited in this study. Circles mark museum collections records with GPS coordinates retrieved from Fishnet2 (http://www.fishnet2.net/). Black outlined circles mark the collection localities of specimens used in this study. The type locality of *Micropterus nigricans* and *Micropterus salmoides* are marked with red-bordered stars. Maps created using personal R scripts. See Supplementary Table S1 for a complete list of specimens used in this study.

We analyzed 394 specimens that allowed representation of all recognized species and hypothesized undescribed species of *Micropterus* throughout their geographic ranges (Fig. 1 and Supplementary Table S1). Our sampling includes specimens collected by the authors and colleagues between 2002 and 2020, and tissue samples provided by museums and state agencies. Field-collected specimens were euthanized using MS-222 and the right-side pectoral fin was removed to provide tissue for DNA extraction. Fin-clips were stored in 100% non-denatured ethanol and the whole-body specimens were fixed in 10% formalin before being transferred to 70% ethanol for long-term preservation. Fin-clips and voucher specimens were cataloged and deposited at the Yale Fish Tissue Collection (YFTC) and Yale Peabody Museum of Natural History (YPM), respectively. All handling of animals was carried out in accordance with, and experimental protocols approved by, Yale University IACUC (No. 2018-10681) and our collecting permits. Our methods conform to ARRIVE guidelines. We obtained additional specimens for DNA extraction from several museum collections and government wildlife agencies (Supplementary Table S1).

The preparation of ddRAD libraries followed protocols outlined in Peterson et al.^44^, using *PstI*/*MspI* restriction enzymes. The details of genomic DNA extraction, ddRAD library preparation, and sequencing are described in Kim et al.^12^. The demultiplexed reads were analyzed with ipyrad v.0.9.68^45^, using default setting with the following exceptions: ‘denovo’ for assembly method, ‘ddrad’ for datatype, ‘TGCAG, CCG’ for restriction overhang, and 0.96 for clustering threshold^46^. The minimum number of specimens sharing a locus, hereafter referred to as ‘min’, was set to various numbers depending on the specific analysis.

### Phylogenomics and population structure analysis

Phylogenies were inferred with maximum likelihood using IQ-TREE 2^47^ from the dataset of concatenated ddRAD loci (min300; 51,717 loci; ~ 16.5% missing portion in data matrix) for 394 individuals of *Micropterus*. The best-fit sequence substitution model (TVM + F + R7) was determined using ModelFinder^48^ implemented in IQ-TREE based on the Bayesian information criterion (BIC). During the IQ-TREE analysis, trees were rooted with the Suwannee Bass (*M. notius*), following its placement in the centrarchid species tree inferred from the dataset of 16 Sanger-sequenced nuclear loci and in preliminary concatenated ddRAD loci phylogenies of *Micropterus* and its sister lineage *Lepomis*^12,49^. We assessed topological support with 1000 ultrafast bootstrap replications^50^.

We performed a population structure analysis with a sparse non-negative matrix factorization algorithm using the ‘snmf’ function implemented in the R package LEA v.3.0.0^51^. We reduced the dataset to only include those 356 specimens for which the missing data comprised less than 20% of the total retained loci. During the snmf analysis, we searched for the optimal number of ancestral populations (K) based on the cross-entropy criterion^52^, through a wide range (K = 1–40) with 10 replications for each scenario on a genotype data matrix for only biallelic, unlinked SNPs (9486 SNPs). With the R package Hierfstat^53^, we estimated Nei’s standard genetic distance (*Ds*) and fixation index (*Fst*) for all pairs among the 19 major lineages identified in the IQ-TREE analysis.

### Species tree and divergence time analyses

We inferred a species-tree using the multispecies coalescent framework using SVDQuartets^54^ as implemented in PAUP* v.4.0a168. Because this implementation of the multispecies coalescent model assumes no gene flow among species^55^, we excluded individuals tentatively identified as having admixed ancestry in the snmf population structure analysis. We treated each of the 19 major IQ-TREE lineages of *Micropterus* as species and subsampled four specimens from geographically spaced sampling locations for each of these species. The dataset was reduced to include 234,131 unlinked SNPs for 76 individuals. We used an exhaustive quartet sampling, standard 1000 bootstrap replicates, and a multispecies coalescent tree model.

We estimated divergence times among the 19 lineages of *Micropterus* using a relaxed molecular clock method in BEAST v.2.5.2^56^. The tree topology was fixed to the SVDQuartets species-tree for the relaxed clock analysis and the taxon sampling was reduced to one specimen per lineage (min19; 9920 loci; ~ 1.8% missing portion in data matrix). We applied an age prior with a normal distribution (mean, 3.11 Mya; SD, 1.3; offset, 5.0) to MRCA of the Suwannee Bass and all other lineages of *Micropterus* derived from Near and Kim (2021) [8.11 Mya; 95% highest posterior density (HPD): 5.84–10.26 Mya]. Two independent runs of 10 million generations were performed, sampling parameters every 2000 generations. Tracer v.1.7.1^57^ was used to check stationarity, to determine the number of generations discarded as burnin, and to confirm that effective sample sizes (ESS) were over 200. The converged runs were combined with LogCombiner (https://www.beast2.org/programs/). Post-burnin tree samples were annotated for mean height using TreeAnnotator (https://www.beast2.org/programs) and the final trees were visualized in FigTree v.1.3.1 (http://tree.bio.ed.ac.uk/software/figtree).

### Genomic species delimitation

We tested hypotheses of species delimitation using the genealogical divergence index (*gdi*)^58^ for each of the four major species complexes, including the Largemouth Bass complex (*Micropterus salmoides* and *M. floridanus*), the Smallmouth Bass complex (*M. dolomieu dolomieu*, *M. dolomieu velox*, *M.* cf. *dolomieu* Little, and *M.* cf. *dolomieu* Ouachita), the Spotted Bass complex (*M. punctulatus*, *M. cf. punctulatus* Choctaw Bass, *M. henshalli*, and *M. treculii*), and the Redeye Bass complex (*M. coosae*, *M. cahabae*, *M. chattahoochae*, *M. cf. coosae* Altamaha Bass, *M. cf. coosae* Bartram’s Bass, *M. tallapoosae*, and *M. warriorensis*). The *gdi* values were calculated using theta and tau parameter estimates resulting from the algorithm A00 of BPP v.4.1^59^. We conducted a BPP analysis using four to ten specimens from geographically spaced sampling locations for each species complex in *Micropterus*, excluding individuals identified as those exhibiting signatures of gene flow in the snmf population structure analysis. The dataset for each species complex contained loci that are shared by all specimens (409–9819 loci). With the R package PopGenome^60^, we estimated average nucleotide diversity within and between species^61^ as a basis for the theta and tau priors. The species tree inferred from the SVDQuartets analysis was used as the guide species tree. For each species complex, we performed three to nine independent runs each for 150,000 generations, sampling parameters every 50 generations, with a burnin of 50,000 generations. Tracer was used to check stationarity and to confirm that ESS were over 200.

### Population genomics and demographic analyses

The snmf population structure analysis identified populations of genetic admixture among the major lineages in the Largemouth Bass and Spotted Bass complexes. To investigate patterns of gene flow between species, we performed population genomic and demographic analyses aimed at the Largemouth Bass and Spotted Bass complexes. For the Largemouth Bass complex, a dataset consisting of 143 specimens of Largemouth Bass and Florida Bass was used to visualize genetic differences among the populations using a PCA implemented in ipyrad. For the PCA, we used a sample imputation method, 50% for ‘minmap’, and 90% for ‘mincov’ (35,270 unlinked SNPs after filtering). With the R package Hierfstat, we calculated pairwise *Fst* values between the sampled populations of Largemouth Bass and Florida Bass and among populations within each of the two species. To estimate directionality and relative magnitude of gene flow between Largemouth Bass and Florida Bass, we used TreeMix v.1.13^62^ implemented in ipyrad. From the dataset used in the PCA, we subsampled unlinked SNPs that are shared by at least 50% of the specimens in each of the populations. In the TreeMix analysis, we inferred the maximum likelihood tree of the lineages with a migration edge of zero and then compared the likelihoods of the migration edges up to 10. We identified the point where log-likelihood values begin to plateau, indicating the optimal number of migration edges.

Using TreeMix with the same settings as the analysis of the Largemouth Bass complex, we identified the patterns of gene flow among major lineages of the Spotted Bass complex that are resolved as well-supported clades in the phylogenetic analysis of the concatenated dataset. Because there is inconsistency in the relationships inferred from the concatenated, species-tree, and TreeMix analyses of the Spotted Bass complex, we additionally performed demographic model testing analyses with Fastsimcoal2 v.2.6^63,64^ at two hierarchical levels. We first tested phylogenetic relationships of the populations in the absence of gene flow. We then added gene flow scenarios to a model that was identified as the best-fit model lacking gene flow. We converted each dataset in the variant call format to a multisite frequency spectrum with the python script derived from Marques et al.^65^; available at: https://github.com/marqueda/SFS-scripts, maximizing the number of segregating sites. Timing of initial population splits were constrained with mean divergence-time estimates inferred from a molecular clock BEAST2 analysis. We assumed a generation time of three years for species of *Micropterus*^66,67^. A log-uniform prior was used for effective population size parameters (1,000–500,000) and migration rate parameters (0–1.0E-9). We estimated parameters 50 times for each model per dataset, determined the best-fit model under Akaike information criterion (AIC), and then obtained 95% confidence intervals for parameters from 100 parametric bootstrap replications of the best-fit model.

## Results and discussion

### Phylogenomics: relationships among species of Micropterus

**Figure 2:**
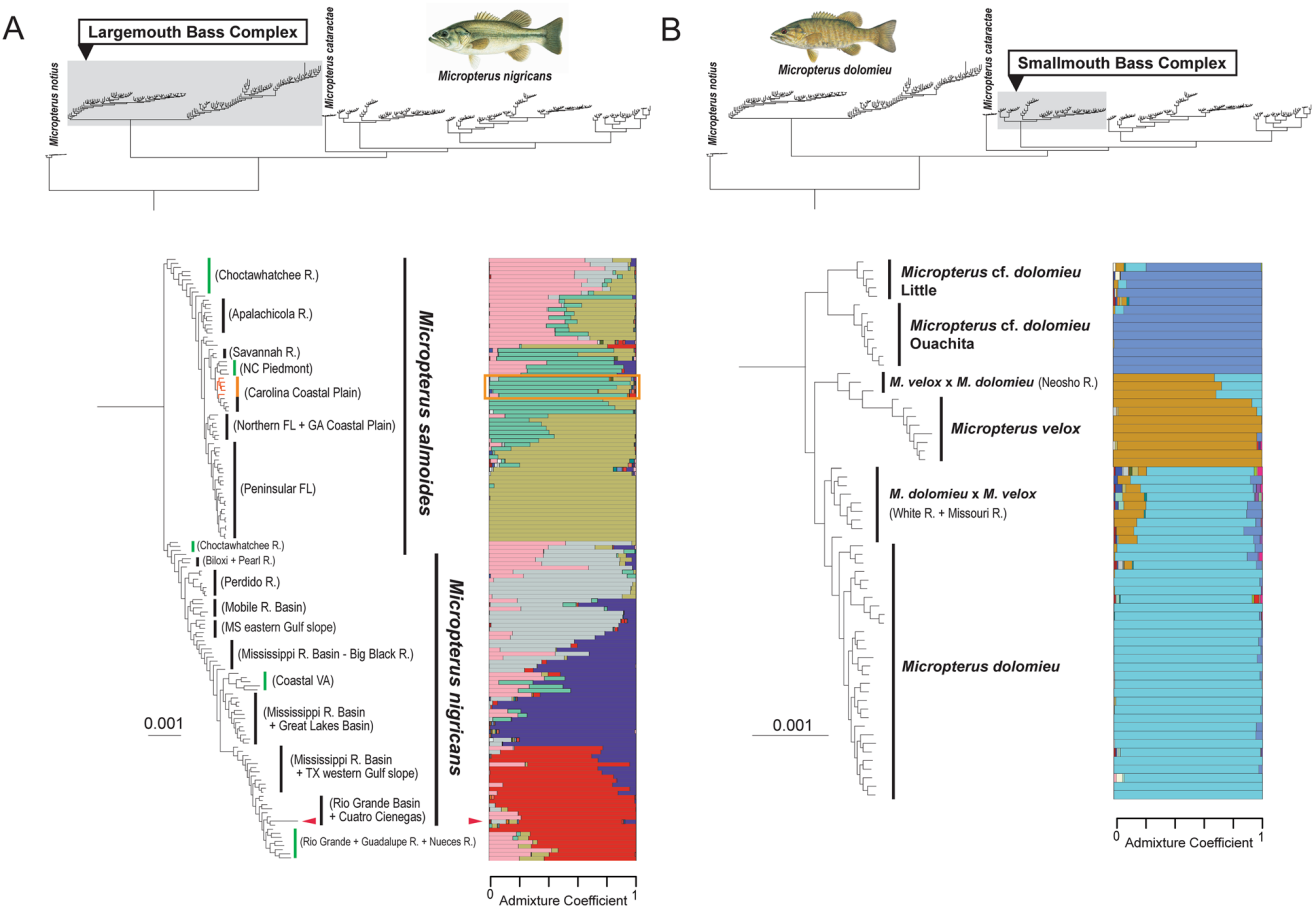
Maximum likelihood phylogeny inferred from IQ-TREE analysis of concatenated ddRAD dataset for (**a**) the Largemouth Bass complex and (**b**) the Smallmouth Bass complex. Population structure (K = 20) inferred from snmf analysis for each species complex are shown to the right of the phylogeny. In the clade delimiting the Largemouth Bass complex (**a**), population structure assignments and branches leading to specimens from the vicinity of Charleston, South Carolina (type locality of *Micropterus salmoides*) are highlighted in orange. Green vertical bars indicate populations of genetic admixture between *M. nigricans* and *M. salmoides* that are identified in TreeMix analysis. Red arrows indicate the phylogenetic placement and population structure assignment of a specimen sampled from Cuatro Ciénegas. See Supplementary Fig. S1 for a completely annotated phylogeny tip labels and bootstrap supports values. Illustrations © Joseph R. Tomelleri, used with permission.

**Figure 3:**
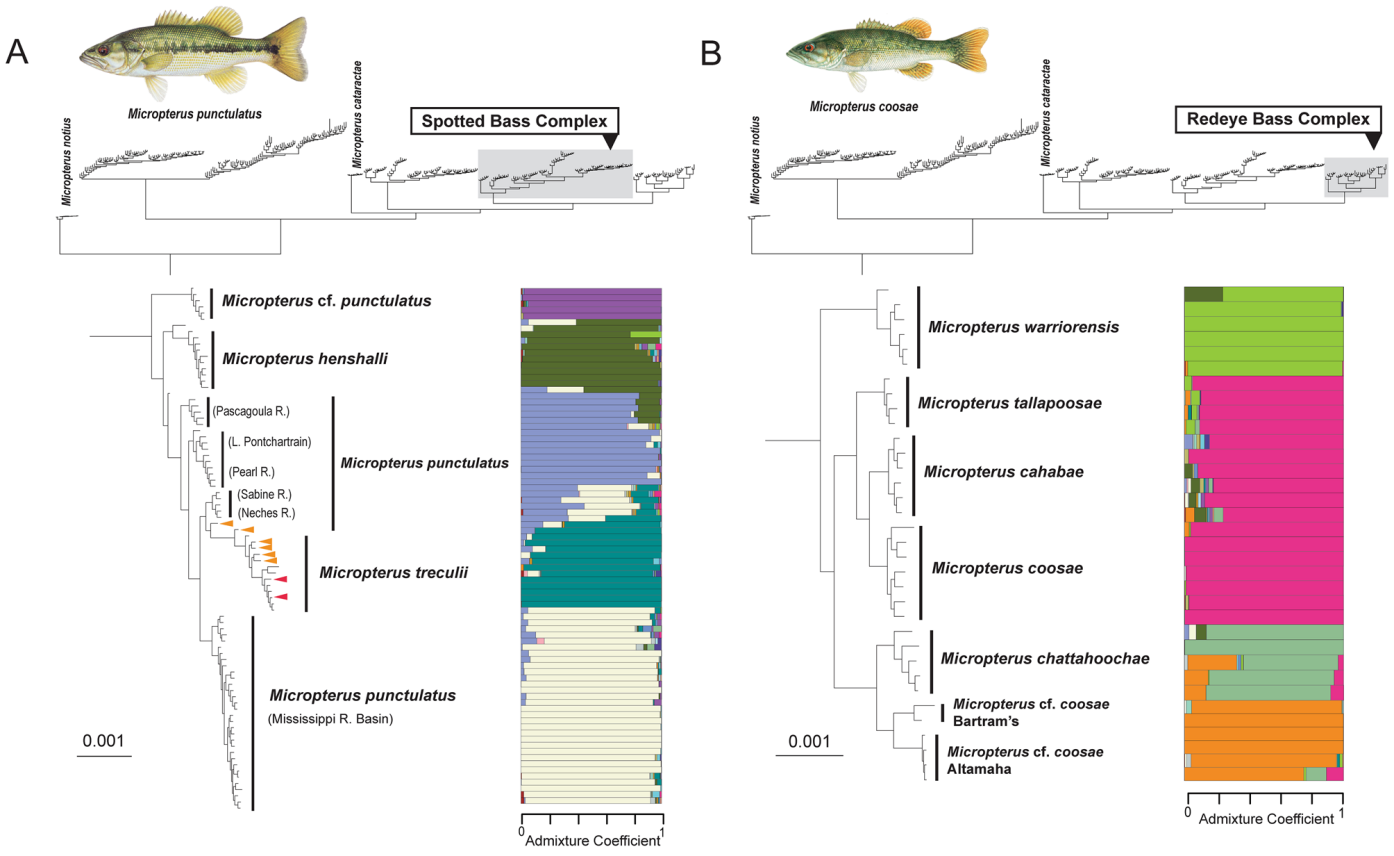
Maximum likelihood phylogeny inferred from IQ-TREE analysis of concatenated ddRAD dataset for (**a**) the Spotted Bass complex and (**b**) the Redeye Bass complex. Population structure (K = 20) inferred from snmf analysis for each species complex are re shown to the right of the phylogeny. In the clade delimiting the Spotted Bass complex (**a**), orange and red arrows indicate specimens from the Brazos and Colorado rivers, respectively, that are identified morphologically as *Micropterus punctulatus*. See Supplementary Fig. S1 for a completely annotated phylogeny tip labels and bootstrap supports values. Illustrations © Joseph R. Tomelleri, used with permission.

Maximum likelihood analysis of the concatenated ddRAD loci dataset using IQ-TREE strongly resolves each of morphologically defined species-complexes as monophyletic groups, places the Suwannee Bass (*Micropterus notius*) as the sister lineage of all other species of *Micropterus*, and resolves the Spotted Bass complex and Redeye Bass complex as sister lineages (Figs. 2, 3; Supplementary Fig. S1). There are two monophyletic groups within the Largemouth Bass complex that correspond to the current delimitation of the Largemouth Bass and the Florida Bass; however, specimens sampled from the type locality of *Micropterus salmoides* are phylogenetically nested well within the lineage currently delimited as the Florida Bass (Figs. 1, 2a), which we now refer to as *Micropterus salmoides*. Greater detail is provided below, but we now apply *Micropterus nigricans* Cuvier^68^ as the scientific name for the Largemouth Bass.

The pairwise *Fst* and Nei’s *Ds* values among the 19 lineages of *Micropterus* were highest in all of the contrasts involving the Suwannee Bass and lowest among the more recently diverged sister lineages. The lowest pairwise *Fst* (0.24) is between the Cahaba Bass and Redeye Bass, while the highest (0.96) is between the Altamaha Bass and Suwannee Bass (Supplementary Table S2). Similarly, the Cahaba Bass and Redeye Bass exhibit the lowest pairwise sequence divergence (*Ds* = 0.01) and the highest pairwise genetic divergence is between the Florida Bass and Suwannee Bass (*Ds* = 0.12; Supplementary Table S2).

### Species tree inference, relaxed molecular clock divergence time estimates, and genomic species delimitation

**Figure 4:**
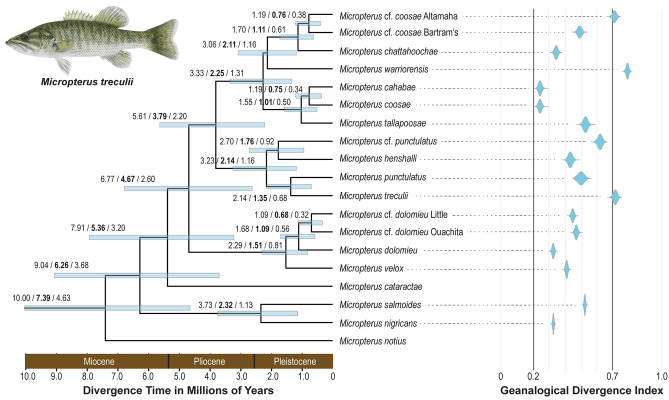
Time-calibrated species tree for *Micropterus*, inferred from SVDQuartets and BEAST2. All nodes have bootstrap supports of 100%, except the node leading to *M. cataractae* and the Smallmouth, Spotted, and Redeye Bass complexes, to *M. warriorensis*, *M. chattahoochae*, and the undescribed Altamaha and Bartram’s basses, and to *M. cahabae* and *M. coosae*. Numbers at nodes indicate a mean and 95% highest posterior density (also depicted with light blue bars) of relaxed molecular clock divergence times. Violin plots for genealogical divergence index (*gdi*) of species delimitations are aligned with and shown on the right of species tree. Illustration © Joseph R. Tomelleri, used with permission.

The phylogenetic relationships of *Micropterus* are congruent between the concatenated data IQ-TREE analysis and a species tree inferred with SVDQuartets, except for the phylogenetic resolution of the Neosho Bass (*Micropterus velox*), Choctaw Bass (*M*. cf. *punctulatus*), and Warrior Bass (*M. warriorensis*) (Fig. 4). In the concatenated analysis the Neosho Bass is resolved as the sister species of the Smallmouth Bass (*M*. *dolomieu*) (Fig. 2b), while Neosho Bass is the sister lineage to all remaining species of the Smallmouth Bass complex in the species tree analysis (Fig. 4). The Choctaw Bass is resolved as the sister species to the Alabama Bass (*M. henshalli*) in the species tree analysis (Fig. 4), although it is placed as the sister lineage of all other species in the Spotted Bass complex in the concatenated analysis (Fig. 3a). The Warrior Bass is resolved as the sister lineage to all remaining species of the Redeye Bass complex in the concatenated analysis (Fig. 3b); however, in the species tree analysis it is resolved as the sister lineage to a clade containing the non-Mobile species of the Redeye Bass complex (Fig. 4). Under certain conditions of phylogenomic analysis, including the presence of gene flow, elevated substitution rates, or a limited taxon sampling, coalescent methods (e.g., SVDQuartets) are more robust than concatenation methods^69–71^.

The posterior age estimate from the relaxed molecular clock analysis for the most recent common ancestor (MRCA) of *Micropterus* is 7.39 Mya (95% HPD 4.63–10.00 Mya; Fig. 4). The posterior ages of the MRCA of the Largemouth Bass, Spotted Bass, and Redeye Bass complexes are similar, ranging between 2.32 and 2.14 Mya (Fig. 4). The estimated age of the MRCA of the Smallmouth Bass complex is 1.51 Mya (95% HPD: 0.81–2.29 Mya; Fig. 4). The mean ages of the most recent inferred speciation events range between 0.68 and 0.76 Mya (Fig. 4).

The BPP species delimitation analysis results in moderate to high genealogical divergence indices (*gdi*) that between 0.242 and 0.789, supporting the delimitation of 19 species of *Micropterus* (Table 1; Fig. 4). As suggested by Jackson et al.^58^, the *gdi* values estimated for the Warrior Bass, Altamaha Bass (*M*. cf. *coosae*), and Guadalupe Bass (*M*. *treculii*) were consistent with the expectations for distinct species (Fig. 4). The lowest *gdi* values were observed in Cahaba Bass and Redeye Bass (Fig. 4). All other species of *Micropterus*, including the undescribed Smallmouth Bass lineages in Little and Ouachita Rivers, Bartram’s Bass, Altamaha Bass, and Choctaw Bass, exhibit *gdi* values between 0.30 and 0.66 that fall within the range expected of distinct species that are supported with other lines of evidence such as morphological traits, differences in coloration and pigmentation, or behavior (Fig. 4).

**Table 1:** List of delimited species of *Micropterus*. Status indicates if delimitation is different from Taylor et al.^24^.

Common name	Scientific name	Status
Alabama Bass	*Micropterus henshalli* Hubbs & Bailey, 1940	As in Taylor et al.^24^
Altamaha Bass	*Micropterus* cf. *coosae* Altamaha River	Discovered in Freeman et al.^88^
Bartram's Bass	*Micropterus* cf. *coosae* Bartram's	Delimited in Freeman et al.^88^
Cahaba Bass	*Micropterus cahabae* Baker, Johnston & Blanton, 2013	As in Taylor et al.^24^
Chattahoochee Bass	*Micropterus chattahoochae* Baker, Johnston & Blanton, 2013	As in Taylor et al.^24^
Choctaw Bass	*Micropterus* cf. *punctulatus*	Discovered in Tringali et al.^85^
Florida Bass	*Micropterus salmoides* (Lacépède, 1802)	Delimitation changed. Most populations were previously delimited as *M. floridanus*
Guadalupe Bass	*Micropterus treculii* (Vaillant & Bocourt, 1874)	As in Taylor et al.^24^
Largemouth Bass	*Micropterus nigricans* (Cuvier, 1828)	Delimitation changed. Previously delimited at *M. salmoides*, Largemouth Bass
Little River Bass	*Micropterus* cf. *dolomieu* Little River	Discovered in this study
Neosho Bass	*Micropterus velox* Hubbs & Bailey, 1940	Elevated from synonymy with *M. dolomieu*
Ouachita Bass	*Micropterus* cf. *dolomieu* Ouachita River	Discovered in Stark and Echelle^83^
Redeye Bass	*Micropterus coosae* Hubbs & Bailey, 1940	As in Taylor et al.^24^
Shoal Bass	*Micropterus cataractae* Williams & Burgess, 1999	As in Taylor et al.^24^
Smallmouth Bass	*Micropterus dolomieu* Lacépède, 1802	As in Taylor et al.^24^
Spotted Bass	*Micropterus punctulatus* (Rafinesque, 1819)	As in Taylor et al.^24^
Suwannee Bass	*Micropterus notius* Bailey & Hubbs, 1949	As in Taylor et al.^24^
Tallapoosa Bass	*Micropterus tallapoosae* Baker, Johnston & Blanton, 2013	As in Taylor et al.^24^
Warrior Bass	*Micropterus warriorensis* Baker, Johnston & Blanton, 2013	As in Taylor et al.^24^

### Species delimitation of Largemouth Bass and Florida Bass, changes to scientific nomenclature, and geographic patterns of introgression

*Micropterus salmoides* was described by Lacépède^41^^:716,717^ based on an illustration of a specimen collected by Louis Bosc in the vicinity of Charleston, South Carolina^32,72,73^. The lack of type specimens and little detail in Lacépède’s^41^ description led to an 80-year period of nomenclatural confusion with *Micropterus dolomieu*, the Smallmouth Bass^74,75^. The application of the name *Micropterus salmoides* to the Largemouth Bass was suggested by Henshall^22^^:110–132^ and has remained unchanged to the present day. A study of geographic variation in morphological traits resulted in *Cichla floridana* Lesueur^42^ being recognized as the subspecies *Micropterus salmoides floridanus*, which was endemic to peninsular Florida^43^. Based on intermediate meristic traits it was hypothesized that *M*. *s*. *salmoides* and *M*. *s*. *floridanus* were hybridizing in a broad geographic area of intergradation that included nearly all of the Florida Panhandle and all of the rivers in Georgia that drain into the Gulf of Mexico and the Atlantic Ocean^43^^: Map 1^. Kassler et al.^29^ concluded that *Micropterus salmoides* and *M. floridanus* are each distinct species based on morphology, fixed differences at a number of allozyme loci, and deep reciprocal monophyly in mitochondrial gene trees. Subsequent studies recognize the Florida Bass as a distinct species relative to the Largemouth Bass (e.g., Refs.^24,76,77^); however, some authors and the Names of Fishes Committee (jointly hosted by the American Fisheries Society and the American Society of Ichthyologists and Herpetologists) continue to treat the Florida Bass and Largemouth Bass as subspecies (e.g., Refs.^40,78,79^).

Results of our ddRAD phylogenomic analyses do not support the hypothesis that Florida Bass and Largemouth Bass hybridize over a broad area of intergradation, but rather the Florida Bass has a much larger geographic distribution that ranges from peninsular Florida in the south and the Apalachicola River in the west to the Cape Fear River of North Carolina in the north along the Atlantic Coastal Plain (Figs. 1, 2A; Supplementary Figs. S1 and S2). Phylogenetic analysis of the ddRAD data strongly resolves populations from peninsular Florida and rivers in Georgia, South Carolina, and North Carolina that include the Savannah, Edisto, Ashley, Santee, Pee Dee, and Cape Fear Rivers as a monophyletic group that we delimit as the Florida Bass (Fig. 2a). The snmf population structure analysis identifies genetic variation associated with geography among populations of Florida Bass and the delimitation of populations in Georgia, South Carolina, and North Carolina as Florida Bass. The presence of Florida Bass in these areas is not likely result from stocking into areas previously occupied by Largemouth Bass (Figs. 1, 2a; Supplementary Fig. S2).

The identification of populations along the Atlantic Coastal Plain of Georgia, South Carolina, and North Carolina as Florida Bass highlights a need to dramatically revise the scientific nomenclature of both Largemouth Bass and Florida Bass. Bailey and Hubbs^43^ delimited Florida Bass as those populations with high counts and Largemouth Bass as populations with low counts in five meristic traits (e.g., number of scales along the lateral line). Populations just south of Charleston, South Carolina, which is the type locality for *Micropterus salmoides*, exhibit high or intermediate scale counts and were delimited by Bailey and Hubbs^43^^: Map 1^ as Florida Bass or intergrades between Florida Bass and Largemouth Bass. Our genomic analyses identify all specimens from the Ashley and Cooper Rivers that approximate the type locality of *M. salmoides* as conspecific with populations sampled from peninsular Florida and distinct from all other populations identified as Largemouth Bass (Fig. 2a; Supplementary Figs. S1 and S2).

Our results highlight that the scientific name *Micropterus salmoides* has been misapplied for the nearly 75 years following the delimitation of Largemouth Bass and Florida Bass outlined in Bailey and Hubbs^43^. In order to present a classification for species of *Micropterus* that is consistent with their phylogenetic relationships, we conclude that Lacépède’s^41^ description of *Micropterus salmoides* is the accurate and valid scientific name for the Florida Bass (Figs. 1, 2a). *Micropterus floridanus*^42^ which is currently applied to the Florida Bass (e.g., Refs.^24,49^) is a junior synonym of *Micropterus salmoides*^41^. The oldest available scientific name for the Largemouth Bass is *Micropterus nigricans* Cuvier^68^ with the type locality given as Lake Huron where the species is native^31,32,80^.

Both the Largemouth Bass and Florida Bass exhibit geographic population genetic structure. The ddRAD data identifies three genetic groups in Largemouth Bass: a southwestern group, ranging from the Rio Grande system in southern Texas and the Cuatro Ciénegas Basin in Coahuila, Mexico to the south to southwestern tributaries of the Mississippi River (e.g., Red and Canadian Rivers in Oklahoma), a northcentral group throughout the Mississippi River Basin and the Laurentian Great Lakes, and a southeastern group that is consistent with the proposed “Delta Bass^81^” that ranges from the southeastern tributaries of the Mississippi River to the Perdido River system in Alabama and Florida (Fig. 2a; Supplementary Fig. S2). There are three genetic groups in the Florida Bass, including one occurring throughout rivers in Florida and in the Atlantic Coastal Plain in the north to the Savannah River system of Georgia, a western group observed in the Choctawhatchee and Apalachicola River systems and rivers in the Atlantic Piedmont, and a northeastern group, ranging from the Satilla River of Georgia in the south to the Cape Fear River of North Carolina in the north along the Atlantic Coastal Plain (Fig. 2a; Supplementary Fig. S2).

**Figure 5:**
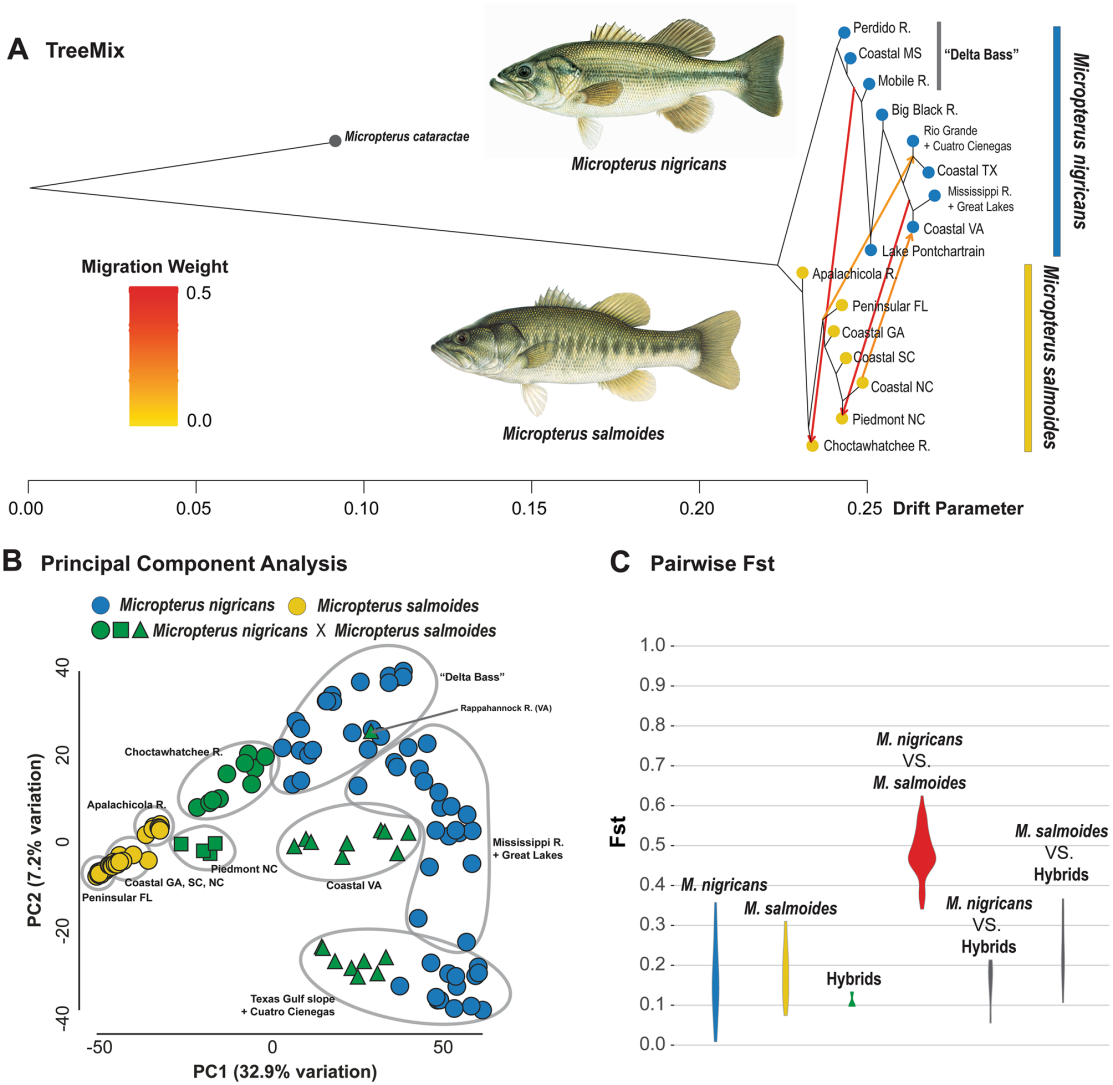
(**a**) Migration edges between *Micropterus nigricans* and *M. salmoides*, inferred from TreeMix analysis. Arrows show relative magnitude and direction of gene flow among populations of the Largemouth Bass complex. (**b**) Principal component analysis (PCA) of genetic variance for species of the Largemouth Bass complex. Grey ellipses indicate major geographic populations or lineages of the Largemouth Bass complex. We separate populations that are genetically admixed between *Micropterus nigricans* and *Micropterus salmoides* by origin of genetic admixture: presumably natural secondary contract (green circles); uncertain (green rectangles); and human-mediated origin (green triangles). (**c**) Violin plots for ranges of pairwise *Fst* values among populations within *Micropterus nigricans*, *M. salmoides*, and genetic admixtures (Choctawhatchee and North Carolina Piedmont) and between each pair of the three groups. Pairwise *Fst* values between populations of *Micropterus nigricans* and *M. salmoides* are considerably higher than other pairs in comparison. Illustrations © Joseph R. Tomelleri, used with permission.

The snmf-inferred population structure and the TreeMix analyses identify signatures of genomic admixture between the Largemouth Bass and Florida Bass from two geographic regions, the Choctawhatchee River in northwestern Florida and rivers draining the Piedmont of North Carolina (Figs. 2a, 5a; Supplementary Fig. S2). Based on analyses of diagnostic nucleotide polymorphisms, we include the Piedmont of Georgia as an area of genomic admixture between Largemouth and Florida Bass^81,82^. Genetic admixture between the two species is also apparent in regions where Florida Bass were introduced to areas where Largemouth Bass are native, including the Rio Grande, Nueces, and Guadalupe River systems in Texas, and several river systems draining into the eastern Gulf of Mexico. Rivers draining into Chesapeake Bay in eastern Virginia also contain admixed individuals where both species were introduced (Figs. 2a, 5a; Supplementary Fig. S2). PCA of genomic diversity in the Largemouth Bass complex reveals the majority of the genetic variation is the result of genetic differentiation between Largemouth Bass and Florida Bass, placing specimens with genomic admixture intermediate along the PC1 axis (Fig. 5b). Pairwise *Fst* values are significantly higher between populations of Largemouth Bass and Florida Bass than those between populations within species or populations of genetic admixture between the two species (Fig. 5c; Supplementary Table S2).

### Species discovery in the Smallmouth Bass complex

Since the description of the Smallmouth Bass by Lacépède^41^, twelve species and a subspecies were described between 1817 and 1940, but all of these scientific names were eventually synonymized with *Micropterus dolomieu*^72,73^. Among the taxa synonymized with the Smallmouth Bass, the Neosho Bass (*M. dolomieu velox*) is the subject of a debate in the systematics of *Micropterus*. Hubbs and Bailey^32^^:29^ described the Neosho Bass as a subspecies of the Smallmouth Bass based on external morphological traits and subsequent analysis observed a significant difference between the Neosho Bass and Smallmouth in head length^33^. The Neosho Bass is limited to a small area of the Arkansas River system, ranging from the Fourche La Fave River upstream to the Neosho River. Hubbs and Bailey^32^^:Map 2^ hypothesized that the White River system of Arkansas and Missouri, the Little River system in Arkansas and Oklahoma, and the upper Ouachita River in Arkansas are areas of intergradation between Neosho Bass and Smallmouth Bass. A range-wide allozyme study of the Smallmouth Bass complex identified several fixed allelic differences consistent with Hubbs and Bailey’s^32^ delimitation of the Neosho Bass and determined the populations of the Smallmouth Bass complex in the Little and Ouachita Rivers are genetically distinct and are not intergrades of Smallmouth Bass and Neosho Bass^83^. Studies using microsatellite loci convincingly identified the Neosho Bass as a distinct lineage and reveal signatures of genetic admixture as the result of human-mediated introductions of non-native Smallmouth Bass^33,34^.

Our phylogenomic analysis of the Smallmouth Bass complex resolves four major lineages: the Smallmouth Bass, the Neosho Bass, an undescribed species we call the Little River Bass (*Micropterus* cf. *dolomieu* Little), and another undescribed species native to the upper Ouachita River system we call the Ouachita Bass (*M*. cf. *dolomieu* Ouachita) (Figs. 1, 2b; Table 1; Supplementary Fig. S1). The BPP analysis resulted in moderate *gdi* values for each four lineages (Fig. 4), supporting the delimitation of each as a distinct species if additional layers of evidence such as phenotype, behavior, or ecology are available^84^. Based on the results of our phylogenomic analyses and documented morphological distinctiveness^32,33^, we elevate the Neosho Bass, *Micropterus velox*, to the species taxonomic rank. Although Stark and Echelle^83^ treated the Little River Bass and Ouachita Bass as single distinct genetic lineage, results from our phylogenomic analyses suggest that the two sister lineages are not only distinct from the Smallmouth Bass and Neosho Bass, they each have a *gdi* value which exceeds that observed for the other two species (Fig. 4). We consider both the Little River Bass and Ouachita Bass as distinct but undescribed species (Table 1).

### The Spotted Bass complex: species delimitation and patterns of introgression

The Spotted Bass complex currently includes Spotted Bass, Guadalupe Bass, Alabama Bass, and the undescribed Choctaw Bass (Table 1). Maximum likelihood analysis of the concatenated ddRAD dataset resolves the Choctaw Bass as the sister lineage of all other species in the Spotted Bass complex (Fig. 3a; Supplementary Fig. S1), the species tree and demographic analyses support a clade containing the Choctaw and Alabama basses (Fig. 4; Supplementary Fig. S3a). The delimitation of both Choctaw Bass and Alabama Bass as distinct species is supported by moderate *gdi* values (Fig. 4) and morphological differences^35,85^.

The Spotted Bass is resolved as paraphyletic in the phylogeny inferred from the concatenated ddRAD dataset as the Guadalupe Bass is the sister lineage of populations sampled from the Sabine and Neches Rivers in Texas and Louisiana (Fig. 3a; Supplementary Fig. S1). Results of the population structure (Fig. 3a) and demographic analyses (Supplementary Fig. S3a,b) indicate that historical introgression from Alabama Bass and Guadalupe Bass may result in the paraphyly of Spotted Bass by resolving introgressed populations outside of a clade containing non-introgressed Spotted Bass plus Guadalupe Bass or as early diverging lineages of the Guadalupe Bass clade. This pattern is consistent with observations from the Longear Sunfish complex (e.g., *Lepomis megalotis*), where introgressed populations are resolved as early diverging or pectinate branches along stem lineages in phylogenetic analyses of concatenated datasets^12^.

Hubbs and Bailey^32^^:Map 1^ hypothesized that populations of Spotted Bass from Lake Pontchartrain, the Pearl River, and the Pascagoula River in Louisiana and Mississippi are intergrades between Spotted Bass and Alabama Bass. In the phylogeny inferred from the concatenated ddRAD dataset, these populations of the Spotted Bass resolve as early diverging lineages in a clade containing all other populations of the Spotted Bass and Guadalupe Bass (Fig. 3a; Supplementary Fig. S1). The Fastsimcoal2 demographic analyses suggests that the MRCA of the Spotted Bass populations from Lake Pontchartrain, the Pearl River, and the Pascagoula River diverged from the MRCA of all other populations of Spotted Bass in the late-Pleistocene. The Fastsimcoal2 and TreeMix analyses identify multiple instances of gene flow from Alabama Bass to the MRCA of the Lake Pontchartrain, the Pearl River, and the Pascagoula River populations (Supplementary Fig. S3a,b). These results provide support for the intergradation hypothesis of Hubbs and Bailey^32^, inferred from morphology. A list of tested models and parameter estimates of each model are presented in Supplementary Fig. S4 and Supplementary Table S3.

The snmf population structure analysis indicates that populations of the Spotted Bass in the Sabine and Neches Rivers in eastern Texas are the result of genetic admixture between the Spotted Bass and Guadalupe Bass (Fig. 3a). These populations of Spotted Bass are resolved as the sister lineage of the Guadalupe Bass in the phylogeny inferred from the concatenated dataset (Fig. 3a; Supplementary Fig. S1); however, the demographic analysis supports a model where the Sabine and Neches populations share common ancestry and are the sister lineage of Mississippi River Basin Spotted Bass populations (Supplementary Fig. S3a). Specimens of the Spotted Bass from the Brazos and Colorado River systems in central Texas are phylogenetically nested within a clade containing Guadalupe Bass in the phylogeny inferred from the concatenated dataset (Fig. 3a; Supplementary Fig. S1). The population structure and demographic analyses suggest specimens of Spotted Bass and Guadalupe Bass from the Brazos River system are genetic admixtures between the two species (Fig. 3a; Supplementary Fig. S3a). Two specimens in our analysis that were identified morphologically as Spotted Bass from the Colorado River system exhibit a genomic ancestry coefficient indicative of Guadalupe Bass (Fig. 3a; Supplementary Fig. S1). This observation is consistent with a study using Sanger-sequenced loci that detected Guadalupe Bass genes in specimens that were phenotypically identified as Spotted Bass^86^, highlighting that mismatch between phenotype and genotype for Spotted and Guadalupe basses in rivers of central Texas will be a challenge for the accurate species identification and conservation management of these populations.

### Redeye Basses: genomic validation of recently described species

Hubbs and Bailey^32^ described the Redeye Bass (*Micropterus coosae*) with a geographic distribution that includes the Mobile Basin eastward to the upper portions of the Apalachicola, Altamaha, Savannah, and Saluda River systems. In a study of external morphology and a mitochondrial gene tree, Baker et al.^87^ described four species from populations of Redeye Bass: the Cahaba Bass (*M. cahabae*) endemic to the Cahaba River system, the Tallapoosa Bass (*M. tallapoosae*) endemic to the Tallapoosa River system, the Warrior Bass (*M. warriorensis*) endemic to the Black Warrior River system, and the Chattahoochee Bass (*M. chattahoochae*) endemic to the Chattahoochee River system. Baker et al.^87^ treated the Bartram’s Bass (*M.* cf*. coosae*) as a distinct and undescribed species distributed in the Savannah River system of Georgia and South Carolina. A subsequent study using morphological traits, DNA sequences from a single nuclear gene, and a mitochondrial gene tree delimited populations of the Redeye Bass complex in the Altamaha and Ogeechee River systems as a distinct and second undescribed species, the Altamaha Bass^88^.

Previous molecular phylogenetic studies of *Micropterus* prior to Near and Kim^49^ did not resolve the Redeye Bass complex as a clade^88–90^, as mitochondrial introgression resulted in an inference of paraphyly of the species comprising the Redeye Bass complex. The phylogenomic analyses of the ddRAD loci resolves the Redeye Bass complex as a monophyletic group with strong node supports and the sampled individuals from every species in the complex resolves as a reciprocally monophyletic group (Fig. 3b; Supplementary Fig. S1). The BPP species delimitation analyses resulted in moderate to high *gdi* values for the species in the Redeye Bass complex, ranging from the lowest among all species in *Micropterus* (0.20–0.27 for Cahaba Bass and Redeye Bass, Fig. 4) to values well within those expected among distinct species for the Altamaha Bass and Warrior Bass (Fig. 4). Given the reciprocal monophyly of all species of the Redeye Bass complex, the documented morphological differences, and the magnitude of genealogical divergence (Fig. 4), we consider the five described and the two undescribed species of the Redeye Bass complex as distinct evolutionary lineages.

## Conclusions

The understanding of *Micropterus* phylogeny and species diversity have dramatically increased over the past 20 years with the application of molecular data and a reexamination of morphological differences^29,33,35,83,85,87,88,91^. The results of our phylogenomic analyses using ddRAD data result in the delimitation of 19 species of *Micropterus* (Fig. 4; Table 1), which includes 14 described species, two undescribed but well-known species in the Redeye Bass complex (Bartram’s Bass and Altamaha Bass), one undescribed but well-known species in the Spotted Bass complex (Choctaw Bass), and the recognition of two undescribed species currently classified as Smallmouth Bass (Little River Bass and Ouachita Bass).

Phylogeographic analysis of ddRAD data results in a revised distribution of the Largemouth Bass, Florida Bass, and hypothesized admixed populations of the two species. The native range of the Florida Bass spans from peninsular Florida northwards to North Carolina along the Atlantic Coastal Plain, including the type locality of *Micropterus salmoides,* which is currently recognized as the scientific name for the Largemouth Bass. Consequently, we synonymize *Micropterus floridanus* with *Micropterus salmoides,* which is retained for the Florida Bass, and elevate *Micropterus nigricans* out of synonymy with *Micropterus salmoides* as the valid scientific name for the Largemouth Bass. We delimit four genetically and geographically distinct evolutionary lineages in the Smallmouth Bass complex, elevating *Micropterus velox* (Neosho Bass) as a species and highlighting the distinctiveness of two undescribed species *M*. cf. *dolomieu* Little (Little River Bass) and *M*. cf. *dolomieu* Ouachita (Ouachita Bass). The phylogenomic analyses reveal a complicated history of interspecific introgression among species of the Spotted Bass complex. The species delimitation analyses support the recognition of seven species in the recently expanded Redeye Bass complex (Table 1).

The genomic based species delimitation outlined in our study provide not only novel understanding of diversity, distribution, and systematics of *Micropterus*, but also an important basis for the management and conservation of economically valuable recreational fishery resources. Human-mediated movement of species and distinct populations across natural boundaries poses one of the greatest threats to the conservation of Black Bass species diversity^24^. Although it may not be possible to ameliorate the consequences of past stockings of species of *Micropterus* that date back to the 1800’s, our results provide strong impetus to discontinue activities that ignore species and population boundaries, including stock enhancement programs and illegal interbasin transfers. Resource agencies and other stakeholders should prioritize conservation efforts towards the many narrow-ranged species to best protect of the unique diversity among the species of *Micropterus*.

## Supplementary Information

Supplementary Information 1.

Supplementary Information 2.

## Data Availability

Raw sequence data is available for download from the SRA (https://www.ncbi.nlm.nih.gov/sra/PRJNA796640). Data and supplementary materials are available from the Dryad Digital Repository: https://datadryad.org/stash/share/I4cSjJcrGRUPSI6uIZhivfBReyoCXLSIIDL89a3Qm20.
