# Supplementary material for: Phylogenomics and species delimitation of the economically important Black Basses (Micropterus)
Source: Sci Rep. 2022 Jun 6;12:9113. doi: 10.1038/s41598-022-11743-2 (PMC9170712; doi:10.1038/s41598-022-11743-2)

Supplementary Figures and Tables for:

Phylogenomics and species delimitation of the economically important Black Basses (*Micropterus*)

Daemin Kim, Andrew T. Taylor, and Thomas J. Near

Supplementary Figure S1. Maximum likelihood phylogeny for all species in *Micropterus*, inferred from IQ–TREE analysis of concatenated ddRAD loci dataset. Bootstrap supports are shown next nodes. Figures 2 and 3 are simplified versions of this figure. Illustrations © Joseph R. Tomelleri, used with permission.

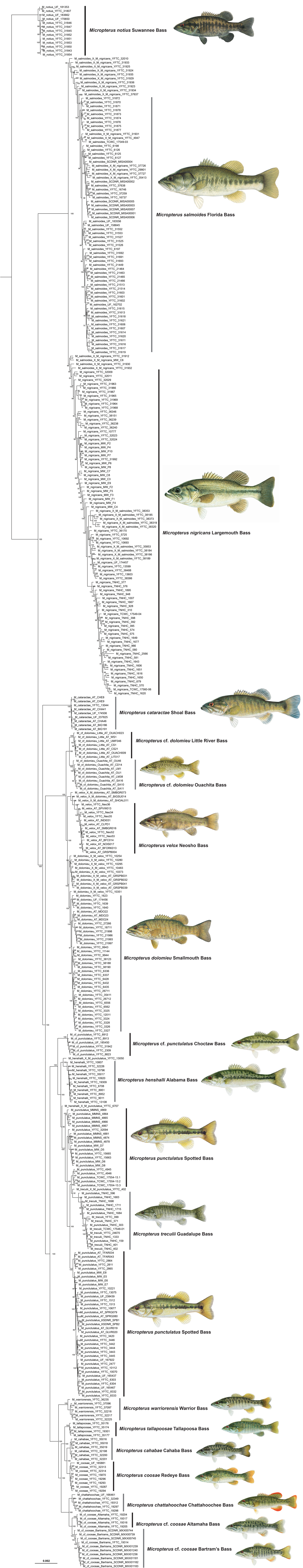

Supplement: Supplementary file 1 — Supplementary Information 1. [file 41598_2022_11743_MOESM1_ESM.pdf]
