# Supplementary material for: Phylogenomics and species delimitation of the economically important Black Basses (Micropterus)
Source: Sci Rep. 2022 Jun 6;12:9113. doi: 10.1038/s41598-022-11743-2 (PMC9170712; doi:10.1038/s41598-022-11743-2)
Supplement: Supplementary file 2 — Supplementary Information 2. [file 41598_2022_11743_MOESM2_ESM.pdf]

Supplementary Figures and Tables for:

Phylogenomics and species delimitation of the economically important Black Basses (*Micropterus*)

Daemin Kim, **Andrew T. Taylor**, and **Thomas J. Near**

Supplementary Figure S2. Map presenting results of snmf analysis for each sampling location for the Largemouth Bass complex. Maps created using personal R scripts.

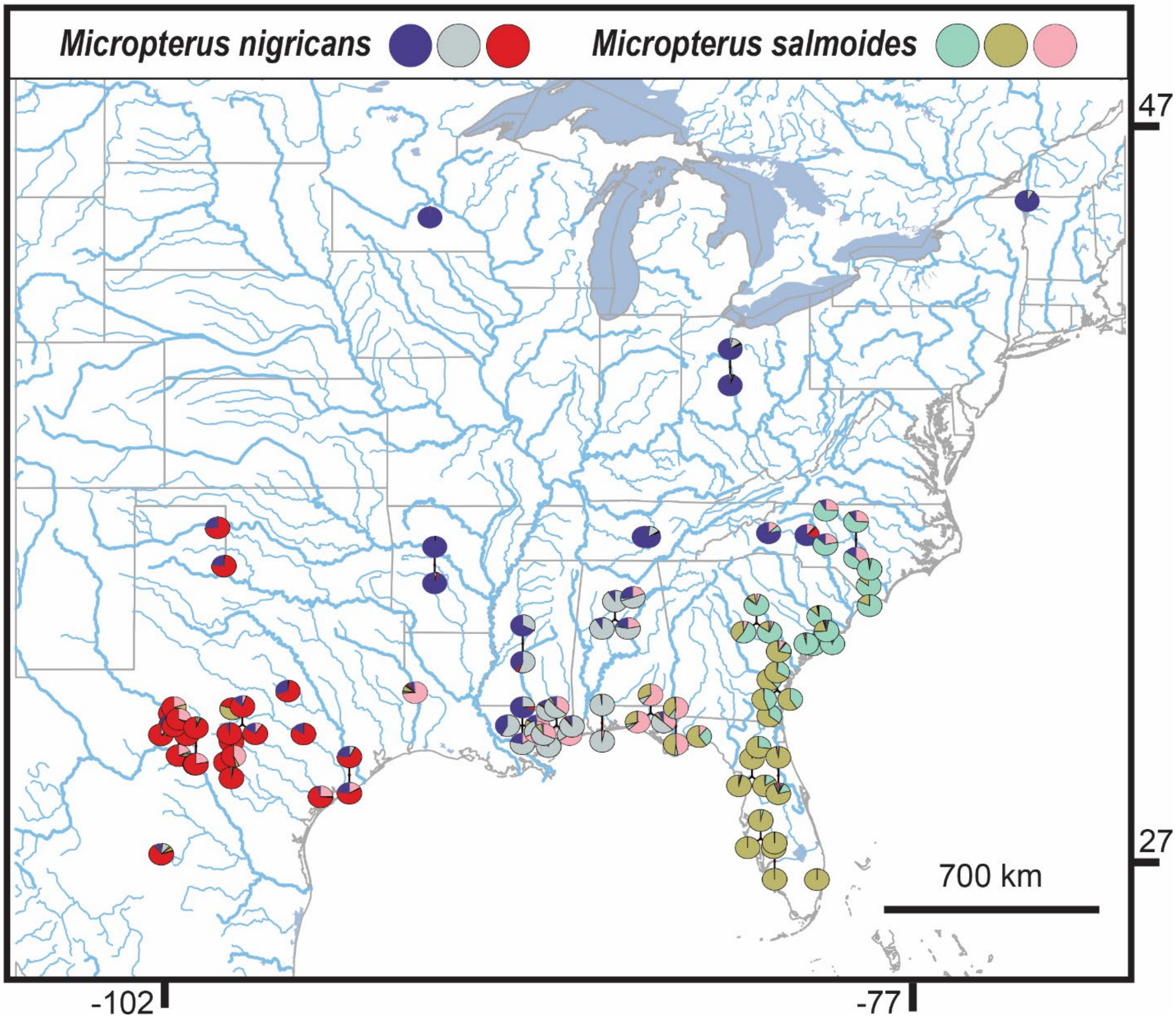

Supplementary Figure S3. (a) Migration edges among populations and species of the Spotted Bass complex, inferred from TreeMix analysis. Arrows show relative magnitude and direction of gene flow. **(b)** Fastsimcoal2 analyses results combined from the models ‘Gene-flow Texas2’ and ‘Gene-flow Spotted_Gulf2’ for the Spotted Bass complex. Phylogenetic tree is trimmed to fit time scales of gene flow. Arrows indicate direction of gene flow. Light blue bars indicate 95% confidence interval (CI) of estimated timing for population split or gene flow events and numbers around light blue bars show a mean and the 95% CI for each event. For details of tested models and parameter estimates, see Supplementary Fig. S4 and Supplementary Table S3.

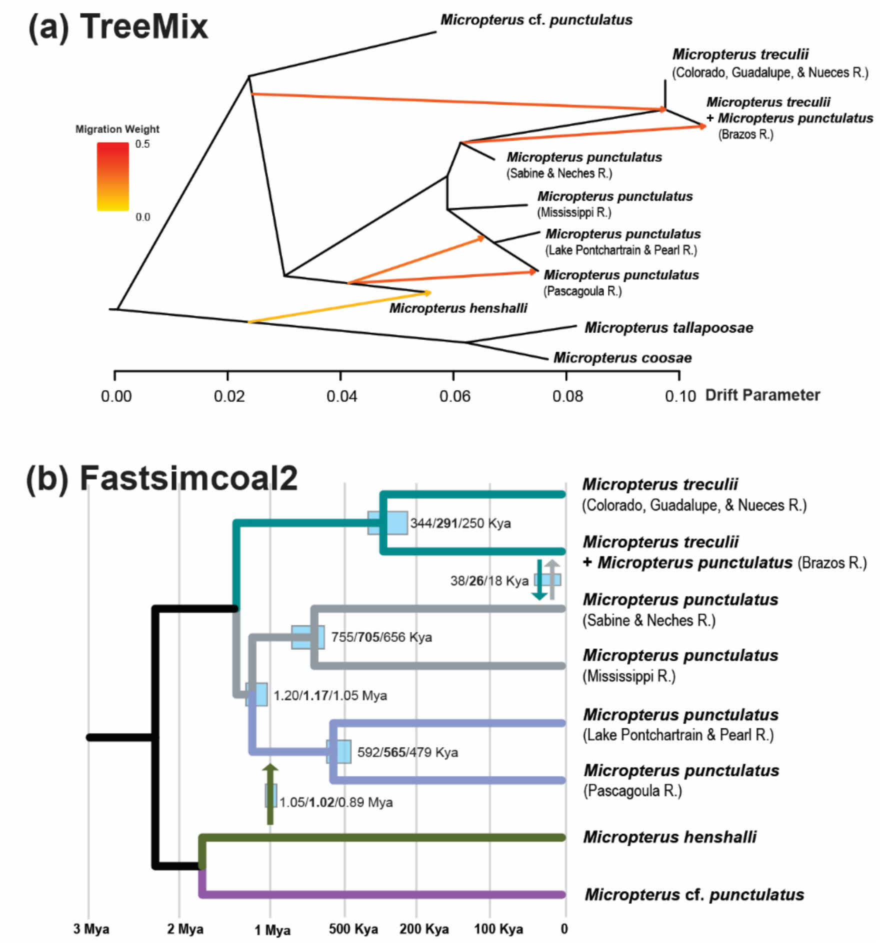

Supplementary Figure S4. Hierarchical models tested in Fastsimcoal2 analyses for the Spotted Bass complex and their parameters. Best-fit model for each set of models is highlighted with a red box. The models ‘Gene-flow Texas2’ and ‘Gene-flow Spotted_Gulf2’ are additionally highlighted with a yellow star. For details of the parameter estimates, see Supplementary Table S3.

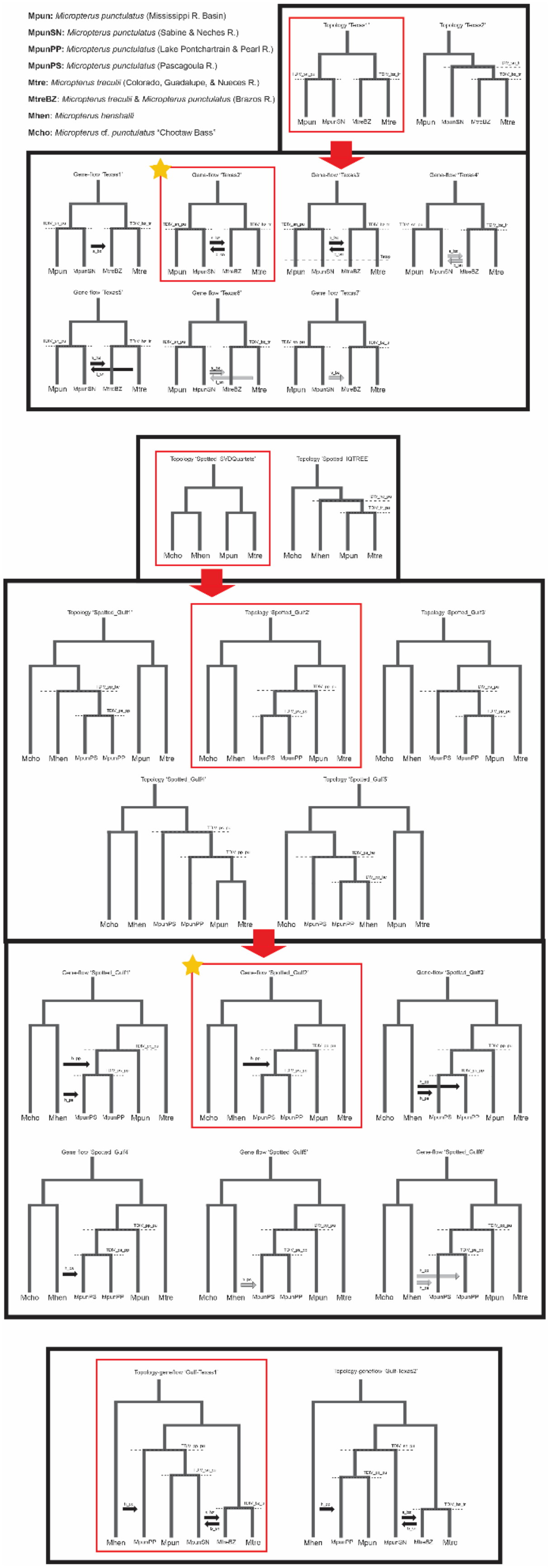
Mpun: *Micropterus punctulatus* (Mississippi R. Basin)
MpunSN: *Micropterus punctulatus* (Sabine & Neches R.)
MpunPP: *Micropterus punctulatus* (Lake Pontchartrain & Pearl R.)
MpunPS: *Micropterus punctulatus* (Pascagoula R.)
Mtre: *Micropterus treculii* (Colorado, Guadalupe, & Nueces R.)
MtreBZ: *Micropterus treculii* & *Micropterus punctulatus* (Brazos R.)
Mhen: *Micropterus henshalli*
Mcho: *Micropterus* cf. *punctulatus* "Choctaw Bass"
Topology 'Texas1'
Topology 'Texas2'
Gene-flow 'Texas1'
Gene-flow 'Texas2'
Gene-flow 'Texas3'
Gene-flow 'Texas4'
Gene-flow 'Texas5'
Gene-flow 'Texas6'
Gene-flow 'Texas7'
Topology 'Spotted_SVDQuartets'
Topology 'Spotted_IQTREE'
Topology 'Spotted_Gulf1'
Topology 'Spotted_Gulf2'
Topology 'Spotted_Gulf3'
Topology 'Spotted_Gulf4'
Topology 'Spotted_Gulf5'
Gene-flow 'Spotted_Gulf1'
Gene-flow 'Spotted_Gulf2'
Gene-flow 'Spotted_Gulf3'
Gene-flow 'Spotted_Gulf4'
Gene-flow 'Spotted_Gulf5'
Gene-flow 'Spotted_Gulf6'
Topology-geneflow 'Gulf-Texas1'
Topology-geneflow 'Gulf-Texas2'
Mpun
MpunSN
MtreBZ
Mtre
Mcho
Mhen
MpunPS
MpunPP

Supplementary Table S1. List of specimens used in the present study, GPS coordinates of sampling locations, and voucher and tissue catalog numbers. Specimen names correspond to IQ-TREE phylogeny shown in Supplementary Fig. S1. Museum and tissue collection catalog abbreviations: GMNHTC, Georgia Museum of Natural History; INHS, Illinois Natural History Survey; MMNS, Mississippi Museum of Natural Science; SELU, Southeastern Louisiana University Vertebrate Museum; TCWC, Biodiversity Research and Teaching Collections (formerly Texas Cooperative Wildlife Collection); TNHC, Texas Natural History Collections; UF, University of Florida, Florida Museum of Natural History; UT, University of Tennessee Etnier Ichthyological Collection; YPM, Yale Peabody Museum of Natural History; AT, gift from Andrew Taylor (University of Central Oklahoma); KS, gift from Ben Neely (Kansas Department of Wildlife, Parks, and Tourism); MW, gift from Matthew Wagner (U.S. Fish and Wildlife Service, formerly Mississippi Museum of Natural Science); SCDNR, South Carolina Department of Natural Resources; YFTC, Yale Fish Tissue Collection. NA, not available.

**Specimen**	**Species**	**Latitude**	**Longitude**	**Voucher**	**Tissue**
M_cahabae_YFTC_35016	*Micropterus cahabae*	33.43989	-86.69921	YPM ICH 033290	YFTC_35016
M_cahabae_YFTC_35018	*Micropterus cahabae*	33.43989	-86.69921	YPM ICH 033290	YFTC_35018
M_cahabae_YFTC_35019	*Micropterus cahabae*	33.43989	-86.69921	YPM ICH 033290	YFTC_35019
M_cahabae_YFTC_32198	*Micropterus cahabae*	32.91110	-87.02787	YPM ICH 031720	YFTC_32198
M_cahabae_YFTC_32200	*Micropterus cahabae*	32.91110	-87.02787	YPM ICH 031720	YFTC_32200
M_cahabae_YFTC_32201	*Micropterus cahabae*	32.91110	-87.02787	YPM ICH 031720	YFTC_32201
M_cataractae_YFTC_13044	*Micropterus cataractae*	32.83909	-84.42204	YPM ICH 020670	YFTC_13044
M_cataractae_UF_237825	*Micropterus cataractae*	30.55246	-85.17089	UF 237825	UF_237825
M_cataractae_AT_BIG186	*Micropterus cataractae*	34.01305	-84.35917	NA	AT_BIG186
M_cataractae_AT_BIG191	*Micropterus cataractae*	34.01305	-84.35917	NA	AT_BIG191
M_cataractae_AT_CHA41	*Micropterus cataractae*	34.56311	-83.62876	NA	AT_CHA41

M_cataractae_AT_CHA46	*Micropterus cataractae*	34.56311	-83.62876	NA	AT_CHA46
M_cataractae_AT_CHE8	*Micropterus cataractae*	34.50422	-83.96885	NA	AT_CHE8
M_cataractae_AT_CHE9	*Micropterus cataractae*	34.50422	-83.96885	NA	AT_CHE9
M_cataractae_UF_174506	*Micropterus cataractae*	31.15836	-84.47909	UF 174506	UF_174506
M_cf_coosae_Altamaha_YFTC_19316	*Micropterus* cf. *coosae* Altamaha	33.74570	-83.46190	GMNHTC 9211	YFTC_19316
M_cf_coosae_Altamaha_YFTC_19317	*Micropterus* cf. *coosae* Altamaha	33.74570	-83.46190	GMNHTC 9213	YFTC_19317
M_cf_coosae_Altamaha_YFTC_19204	*Micropterus* cf. *coosae* Altamaha	33.78650	-83.47390	GMNHTC 10349	YFTC_19204
M_cf_coosae_Altamaha_YFTC_19205	*Micropterus* cf. *coosae* Altamaha	33.78650	-83.47390	GMNHTC 10350	YFTC_19205
M_cf_coosae_Bartrams_SCDNR_MXX00739	*Micropterus* cf. *coosae* Bartram's	34.18204	-83.14680	NA	SCDNR_MXX00739
M_cf_coosae_Bartrams_SCDNR_MXX00744	*Micropterus* cf. *coosae* Bartram's	34.18204	-83.14680	NA	SCDNR_MXX00744
M_cf_coosae_Bartrams_SCDNR_MXX00745	*Micropterus* cf. *coosae* Bartram's	34.18204	-83.14680	NA	SCDNR_MXX00745
M_cf_coosae_Bartrams_SCDNR_MXX01181	*Micropterus* cf. *coosae* Bartram's	34.08821	-82.32096	NA	SCDNR_MXX01181
M_cf_coosae_Bartrams_SCDNR_MXX01182	*Micropterus* cf. *coosae* Bartram's	34.08821	-82.32096	NA	SCDNR_MXX01182
M_cf_coosae_Bartrams_SCDNR_MXX01183	*Micropterus* cf. *coosae* Bartram's	34.08821	-82.32096	NA	SCDNR_MXX01183
M_cf_coosae_Bartrams_SCDNR_MXX01239	*Micropterus* cf. *coosae* Bartram's	33.51394	-81.99637	NA	SCDNR_MXX01239
M_cf_coosae_Bartrams_SCDNR_MXX01240	*Micropterus* cf. *coosae* Bartram's	33.51394	-81.99637	NA	SCDNR_MXX01240
M_cf_coosae_Bartrams_SCDNR_MXX01236	*Micropterus* cf. *coosae* Bartram's	34.31152	-82.61862	NA	SCDNR_MXX01236
M_cf_coosae_Bartrams_YFTC_19314	*Micropterus* cf. *coosae* Bartram's	34.03120	-83.00450	GMNHTC 10345	YFTC_19314
M_cf_dolomieu_Little_AT_CS1	*Micropterus* cf. *dolomieu* Little	34.29647	-94.18485	NA	AT_CS1
M_cf_dolomieu_Little_AT_CS21	*Micropterus* cf. *dolomieu* Little	34.29647	-94.18485	NA	AT_CS21
M_cf_dolomieu_Little_AT_WS1	*Micropterus* cf. *dolomieu* Little	34.20877	-94.05485	NA	AT_WS1
M_cf_dolomieu_Little_AT_OUACH009	*Micropterus* cf. *dolomieu* Little	34.16870	-94.91279	NA	AT_OUACH009
M_cf_dolomieu_Little_AT_OUACH023	*Micropterus* cf. *dolomieu* Little	34.06568	-94.62309	NA	AT_OUACH023
M_cf_dolomieu_Little_AT_LIT017	*Micropterus* cf. *dolomieu* Little	34.55663	-94.94042	NA	AT_LIT017

M_cf_dolomieu_Little_AT_UMF046	*Micropterus* cf. *dolomieu* Little	34.45999	-94.63709	NA	AT_UMF046
M_cf_dolomieu_Ouachita_AT_CD14	*Micropterus* cf. *dolomieu* Ouachita	34.39751	-93.62186	NA	AT_CD14
M_cf_dolomieu_Ouachita_AT_SA10	*Micropterus* cf. *dolomieu* Ouachita	34.61309	-92.78072	NA	AT_SA10
M_cf_dolomieu_Ouachita_AT_SA11	*Micropterus* cf. *dolomieu* Ouachita	34.61309	-92.78072	NA	AT_SA11
M_cf_dolomieu_Ouachita_AT_SA16	*Micropterus* cf. *dolomieu* Ouachita	34.61309	-92.78072	NA	AT_SA16
M_cf_dolomieu_Ouachita_AT_LM1	*Micropterus* cf. *dolomieu* Ouachita	34.30479	-93.89525	NA	AT_LM1
M_cf_dolomieu_Ouachita_AT_LM38	*Micropterus* cf. *dolomieu* Ouachita	34.10213	-93.71556	NA	AT_LM38
M_cf_dolomieu_Ouachita_AT_OU1	*Micropterus* cf. *dolomieu* Ouachita	34.61343	-93.69837	NA	AT_OU1
M_cf_dolomieu_Ouachita_AT_OU46	*Micropterus* cf. *dolomieu* Ouachita	34.57158	-93.99355	NA	AT_OU46
M_cf_punctulatus_YFTC_8912	*Micropterus* cf. *punctulatus*	31.12968	-87.08745	YPM ICH 016101	YFTC_8912
M_cf_punctulatus_YFTC_2309	*Micropterus* cf. *punctulatus*	30.92468	-86.55987	NA	YFTC_2309
M_cf_punctulatus_YFTC_8913	*Micropterus* cf. *punctulatus*	31.12968	-87.08745	YPM ICH 016101	YFTC_8913
M_cf_punctulatus_YFTC_31942	*Micropterus* cf. *punctulatus*	30.72443	-86.79291	YPM ICH 031429	YFTC_31942
M_cf_punctulatus_UF_185450	*Micropterus* cf. *punctulatus*	30.98400	-87.23500	UF 185450	UF_185450
M_cf_punctulatus_YFTC_8823	*Micropterus* cf. *punctulatus*	30.92538	-86.55927	YPM ICH 016043	YFTC_8823
M_chattahoochae_YFTC_19297	*Micropterus chattahoochae*	33.34745	-85.23891	GMNHTC 4893	YFTC_19297
M_chattahoochae_YFTC_19298	*Micropterus chattahoochae*	33.34745	-85.23891	GMNHTC 4894	YFTC_19298
M_chattahoochae_YFTC_19312	*Micropterus chattahoochae*	34.62737	-83.64192	GMNHTC 9217	YFTC_19312
M_chattahoochae_YFTC_32349	*Micropterus chattahoochae*	33.39165	-85.16953	YPM ICH 031731	YFTC_32349
M_chattahoochae_UF_166461	*Micropterus chattahoochae*	33.30528	-85.18722	UF 166461	UF_166461
M_coosae_UF_165881	*Micropterus coosae*	33.62528	-86.04806	UF 165881	UF_165881
M_coosae_YFTC_32313	*Micropterus coosae*	33.72512	-85.60121	YPM ICH 031375	YFTC_32313
M_coosae_YFTC_32314	*Micropterus coosae*	33.72512	-85.60121	YPM ICH 031375	YFTC_32314
M_coosae_YFTC_15870	*Micropterus coosae*	34.41596	-84.36685	YPM ICH 021737	YFTC_15870

M_coosae_YFTC_19287	*Micropterus coosae*	34.70403	-84.53940	GMNHTC 2112	YFTC_19287
M_coosae_YFTC_19293	*Micropterus coosae*	34.98887	-84.63475	GMNHTC 3910	YFTC_19293
M_coosae_YFTC_19294	*Micropterus coosae*	34.88924	-84.82729	GMNHTC 3925	YFTC_19294
M_coosae_YFTC_19296	*Micropterus coosae*	34.02841	-85.61439	GMNHTC 3953	YFTC_19296
M_dolomieu_X_M_velox_AT_GRSPB031	*Micropterus dolomieu*	36.23398	-92.92202	NA	AT_GRSPB031
M_dolomieu_X_M_velox_AT_GRSPB032	*Micropterus dolomieu*	36.23398	-92.92202	NA	AT_GRSPB032
M_dolomieu_X_M_velox_AT_GRSPB041	*Micropterus dolomieu*	35.87345	-93.90916	NA	AT_GRSPB041
M_dolomieu_X_M_velox_AT_GRSPB039	*Micropterus dolomieu*	35.87345	-93.90916	NA	AT_GRSPB039
M_dolomieu_X_M_velox_YFTC_10483	*Micropterus dolomieu*	36.61785	-90.83988	YPM ICH 018619	YFTC_10483
M_dolomieu_X_M_velox_YFTC_10254	*Micropterus dolomieu*	37.60104	-92.23398	YPM ICH 018546	YFTC_10254
M_dolomieu_X_M_velox_YFTC_10280	*Micropterus dolomieu*	37.50395	-92.22902	YPM ICH 018534	YFTC_10280
M_dolomieu_X_M_velox_YFTC_10295	*Micropterus dolomieu*	37.50395	-92.22902	YPM ICH 018534	YFTC_10295
M_dolomieu_YFTC_1639	*Micropterus dolomieu*	41.35553	-88.40391	NA	YFTC_1639
M_dolomieu_YFTC_1640	*Micropterus dolomieu*	41.35553	-88.40391	NA	YFTC_1640
M_dolomieu_YFTC_1623	*Micropterus dolomieu*	41.73703	-89.57386	INHS 52554	YFTC_1623
M_dolomieu_YFTC_6336	*Micropterus dolomieu*	36.69210	-86.34727	UT 90.3719	YFTC_6336
M_dolomieu_YFTC_6337	*Micropterus dolomieu*	36.69210	-86.34727	UT 90.3719	YFTC_6337
M_dolomieu_YFTC_6428	*Micropterus dolomieu*	37.26647	-85.58145	UT 90.3739	YFTC_6428
M_dolomieu_YFTC_6432	*Micropterus dolomieu*	37.26647	-85.58145	UT 90.3739	YFTC_6432
M_dolomieu_YFTC_6435	*Micropterus dolomieu*	37.26647	-85.58145	UT 90.3739	YFTC_6435
M_dolomieu_YFTC_3324	*Micropterus dolomieu*	35.76600	-83.85654	NA	YFTC_3324
M_dolomieu_UF_174456	*Micropterus dolomieu*	44.44423	-93.19098	UF 174456	UF_174456
M_dolomieu_YFTC_35411	*Micropterus dolomieu*	35.43880	-79.99930	YPM ICH 033511	YFTC_35411
M_dolomieu_YFTC_Neo34	*Micropterus dolomieu*	NA	NA	NA	YFTC_Neo34

M_dolomieu_YFTC_Neo35	*Micropterus dolomieu*	NA	NA	NA	YFTC_Neo35
M_dolomieu_YFTC_Neo36	*Micropterus dolomieu*	NA	NA	NA	YFTC_Neo36
M_dolomieu_YFTC_Neo52	*Micropterus dolomieu*	NA	NA	NA	YFTC_Neo52
M_dolomieu_YFTC_Neo53	*Micropterus dolomieu*	NA	NA	NA	YFTC_Neo53
M_dolomieu_X_M_velox_YFTC_10373	*Micropterus dolomieu*	36.75436	-92.15351	YPM ICH 020379	YFTC_10373
M_dolomieu_YFTC_11144	*Micropterus dolomieu*	40.38286	-82.22906	YPM ICH 017292	YFTC_11144
M_dolomieu_X_M_velox_YFTC_10351	*Micropterus dolomieu*	37.69276	-92.95593	YPM ICH 020935	YFTC_10351
M_dolomieu_YFTC_6556	*Micropterus dolomieu*	35.57126	-85.71177	UT 90.3730	YFTC_6556
M_dolomieu_YFTC_6562	*Micropterus dolomieu*	35.57126	-85.71177	UT 90.3756	YFTC_6562
M_dolomieu_YFTC_3325	*Micropterus dolomieu*	35.76600	-83.85654	NA	YFTC_3325
M_dolomieu_YFTC_3326	*Micropterus dolomieu*	35.76600	-83.85654	NA	YFTC_3326
M_dolomieu_YFTC_3327	*Micropterus dolomieu*	35.76600	-83.85654	NA	YFTC_3327
M_dolomieu_YFTC_3328	*Micropterus dolomieu*	35.76600	-83.85654	NA	YFTC_3328
M_dolomieu_YFTC_26711	*Micropterus dolomieu*	36.18016	-82.53411	YPM ICH 026300	YFTC_26711
M_dolomieu_YFTC_26712	*Micropterus dolomieu*	36.18016	-82.53411	YPM ICH 026300	YFTC_26712
M_dolomieu_YFTC_12011	*Micropterus dolomieu*	36.82744	-81.92425	YPM ICH 018799	YFTC_12011
M_dolomieu_YFTC_38188	*Micropterus dolomieu*	38.36938	-77.79953	YPM ICH 033644	YFTC_38188
M_dolomieu_YFTC_38190	*Micropterus dolomieu*	38.36938	-77.79953	YPM ICH 033644	YFTC_38190
M_dolomieu_YFTC_18711	*Micropterus dolomieu*	44.36950	-72.73200	YPM ICH 022033	YFTC_18711
M_dolomieu_YFTC_21986	*Micropterus dolomieu*	44.88170	-73.23220	YPM ICH 030968	YFTC_21986
M_dolomieu_YFTC_21993	*Micropterus dolomieu*	44.88170	-73.23220	YPM ICH 030968	YFTC_21993
M_dolomieu_YFTC_21997	*Micropterus dolomieu*	44.88170	-73.23220	YPM ICH 030968	YFTC_21997
M_dolomieu_YFTC_21998	*Micropterus dolomieu*	44.88170	-73.23220	YPM ICH 030968	YFTC_21998
M_dolomieu_YFTC_27266	*Micropterus dolomieu*	44.56060	-73.31480	YPM ICH 028983	YFTC_27266

M_dolomieu_AT_MDO23	*Micropterus dolomieu*	46.63662	-90.92231	NA	AT_MDO23
M_dolomieu_AT_MDO22	*Micropterus dolomieu*	46.63662	-90.92231	NA	AT_MDO22
M_dolomieu_AT_MDO24	*Micropterus dolomieu*	46.63662	-90.92231	NA	AT_MDO24
M_dolomieu_YFTC_9943	*Micropterus dolomieu*	38.49931	-81.06567	YPM ICH 018372	YFTC_9943
M_dolomieu_YFTC_9944	*Micropterus dolomieu*	38.49931	-81.06567	YPM ICH 018372	YFTC_9944
M_dolomieu_YFTC_38123	*Micropterus dolomieu*	38.92410	-78.84125	YPM ICH 033685	YFTC_38123
M_henshalli_X_M_punctulatus_YFTC_13050	*Micropterus henshalli*	32.83909	-84.42204	YPM ICH 020669	YFTC_13050
M_henshalli_X_M_punctulatus_YFTC_6707	*Micropterus henshalli*	34.99047	-84.77559	UT 90.3827	YFTC_6707
M_henshalli_YFTC_8951	*Micropterus henshalli*	31.99653	-87.06859	YPM ICH 018698	YFTC_8951
M_henshalli_YFTC_8952	*Micropterus henshalli*	31.99653	-87.06859	YPM ICH 018698	YFTC_8952
M_henshalli_YFTC_9011	*Micropterus henshalli*	32.77440	-87.27367	YPM ICH 018352	YFTC_9011
M_henshalli_YFTC_19309	*Micropterus henshalli*	34.67817	-83.68524	GMNHTC 9212	YFTC_19309
M_henshalli_YFTC_6708	*Micropterus henshalli*	34.99047	-84.77559	UT 90.3827	YFTC_6708
M_henshalli_YFTC_35017	*Micropterus henshalli*	33.43989	-86.69921	YPM ICH 033289	YFTC_35017
M_henshalli_YFTC_32226	*Micropterus henshalli*	33.91751	-86.81700	YPM ICH 031737	YFTC_32226
M_henshalli_YFTC_10920	*Micropterus henshalli*	34.28470	-87.39965	YPM ICH 018576	YFTC_10920
M_henshalli_YFTC_13108	*Micropterus henshalli*	33.06649	-85.88009	YPM ICH 021060	YFTC_13108
M_henshalli_YFTC_10807	*Micropterus henshalli*	33.79018	-88.31517	YPM ICH 020447	YFTC_10807
M_henshalli_YFTC_10796	*Micropterus henshalli*	33.79018	-88.31517	YPM ICH 020447	YFTC_10796
M_nigricans_YFTC_36346	*Micropterus nigricans*	33.98471	-86.42481	YPM ICH 031988	YFTC_36346
M_nigricans_YFTC_36238	*Micropterus nigricans*	33.37925	-87.01844	YPM ICH 032190	YFTC_36238
M_nigricans_YFTC_36239	*Micropterus nigricans*	33.37925	-87.01844	YPM ICH 032190	YFTC_36239
M_nigricans_YFTC_36240	*Micropterus nigricans*	33.37925	-87.01844	YPM ICH 032190	YFTC_36240
M_nigricans_YFTC_10692	*Micropterus nigricans*	34.87406	-93.03207	YPM ICH 017507	YFTC_10692

M_nigricans_YFTC_10693	*Micropterus nigricans*	34.87406	-93.03207	YPM ICH 017507	YFTC_10693
M_nigricans_YFTC_5725	*Micropterus nigricans*	35.64413	-85.89994	UT 90.3664	YFTC_5725
M_nigricans_YFTC_31963	*Micropterus nigricans*	30.52303	-87.44564	YPM ICH 031634	YFTC_31963
M_nigricans_YFTC_31964	*Micropterus nigricans*	30.52303	-87.44564	YPM ICH 031634	YFTC_31964
M_nigricans_YFTC_31965	*Micropterus nigricans*	30.52303	-87.44564	YPM ICH 031634	YFTC_31965
M_nigricans_YFTC_31966	*Micropterus nigricans*	30.52303	-87.44564	YPM ICH 031634	YFTC_31966
M_nigricans_YFTC_31967	*Micropterus nigricans*	30.52303	-87.44564	YPM ICH 031634	YFTC_31967
M_nigricans_YFTC_31968	*Micropterus nigricans*	30.52303	-87.44564	YPM ICH 031634	YFTC_31968
M_nigricans_YFTC_31969	*Micropterus nigricans*	30.52303	-87.44564	YPM ICH 031634	YFTC_31969
M_nigricans_MW_C3	*Micropterus nigricans*	30.45580	-90.10470	NA	MW_C3
M_nigricans_MW_C4	*Micropterus nigricans*	30.45580	-90.10470	NA	MW_C4
M_nigricans_MW_C6	*Micropterus nigricans*	30.45580	-90.10470	NA	MW_C6
M_nigricans_MW_C7	*Micropterus nigricans*	30.45580	-90.10470	NA	MW_C7
M_nigricans_MW_C8	*Micropterus nigricans*	30.45580	-90.10470	NA	MW_C8
M_nigricans_YFTC_38488	*Micropterus nigricans*	41.43296	-70.66612	YPM ICH 033700	YFTC_38488
M_nigricans_UF_174457	*Micropterus nigricans*	44.44423	-93.19098	UF 174457	UF_174457
M_nigricans_MW_E9	*Micropterus nigricans*	32.70774	-90.09378	NA	MW_E9
M_nigricans_MW_F1	*Micropterus nigricans*	32.70774	-90.09378	NA	MW_F1
M_nigricans_MW_F2	*Micropterus nigricans*	32.70774	-90.09378	NA	MW_F2
M_nigricans_MW_F3	*Micropterus nigricans*	32.70774	-90.09378	NA	MW_F3
M_nigricans_MW_F4	*Micropterus nigricans*	32.70774	-90.09378	NA	MW_F4
M_nigricans_MW_F5	*Micropterus nigricans*	32.70774	-90.09378	NA	MW_F5
M_nigricans_YFTC_32009	*Micropterus nigricans*	30.45973	-88.96561	YPM ICH 031631	YFTC_32009
M_nigricans_YFTC_32010	*Micropterus nigricans*	30.45973	-88.96561	YPM ICH 031631	YFTC_32010

M_nigricans_YFTC_32011	*Micropterus nigricans*	30.45973	-88.96561	YPM ICH 031631	YFTC_32011
M_nigricans_YFTC_31992	*Micropterus nigricans*	30.43636	-88.45230	YPM ICH 031662	YFTC_31992
M_nigricans_MW_P10	*Micropterus nigricans*	30.61002	-88.59009	NA	MW_P10
M_nigricans_MW_P2	*Micropterus nigricans*	30.61002	-88.59009	NA	MW_P2
M_nigricans_MW_P4	*Micropterus nigricans*	30.61002	-88.59009	NA	MW_P4
M_nigricans_MW_P6	*Micropterus nigricans*	30.61002	-88.59009	NA	MW_P6
M_nigricans_MW_P7	*Micropterus nigricans*	30.61002	-88.59009	NA	MW_P7
M_nigricans_MW_P8	*Micropterus nigricans*	30.61002	-88.59009	NA	MW_P8
M_nigricans_YFTC_32029	*Micropterus nigricans*	30.28007	-89.63077	YPM ICH 031513	YFTC_32029
M_nigricans_YFTC_32023	*Micropterus nigricans*	30.37783	-89.23148	YPM ICH 031437	YFTC_32023
M_nigricans_YFTC_32024	*Micropterus nigricans*	30.37783	-89.23148	YPM ICH 031437	YFTC_32024
M_nigricans_TNHC_2566	*Micropterus nigricans*	26.91630	-102.15560	TNHC 26695	TNHC_2566
M_nigricans_YFTC_10777	*Micropterus nigricans*	32.77242	-89.41858	YPM ICH 018888	YFTC_10777
M_nigricans_TCWC_17560-06	*Micropterus nigricans*	29.39166	-101.01980	TCWC 17560-06	TCWC_17560-06
M_nigricans_TCWC_17549-04	*Micropterus nigricans*	30.91892	-99.78395	TCWC 17549-04	TCWC_17549-04
M_nigricans_YFTC_13599	*Micropterus nigricans*	40.32225	-83.17499	NA	YFTC_13599
M_nigricans_YFTC_13603	*Micropterus nigricans*	40.32225	-83.17499	NA	YFTC_13603
M_nigricans_YFTC_36170	*Micropterus nigricans*	35.64744	-86.04372	YPM ICH 031907	YFTC_36170
M_nigricans_TNHC_1895	*Micropterus nigricans*	31.40776	-97.93404	TNHC 65632	TNHC_1895
M_nigricans_TNHC_948	*Micropterus nigricans*	35.90240	-100.27740	TNHC 59372	TNHC_948
M_nigricans_TNHC_376	*Micropterus nigricans*	29.09360	-95.87830	TNHC 47470	TNHC_376
M_nigricans_TNHC_377	*Micropterus nigricans*	29.09360	-95.87830	TNHC 47470	TNHC_377
M_nigricans_TNHC_310	*Micropterus nigricans*	30.22257	-97.40945	TNHC 47933	TNHC_310
M_nigricans_TNHC_392	*Micropterus nigricans*	30.48120	-99.44710	TNHC 47343	TNHC_392

M_nigricans_TNHC_395	*Micropterus nigricans*	30.48120	-99.44710	TNHC 47343	TNHC_395
M_nigricans_TNHC_398	*Micropterus nigricans*	30.48120	-99.44710	TNHC 47343	TNHC_398
M_nigricans_TNHC_580	*Micropterus nigricans*	29.89439	-100.99546	TNHC 55481	TNHC_580
M_nigricans_TNHC_581	*Micropterus nigricans*	29.89439	-100.99546	TNHC 55481	TNHC_581
M_nigricans_TNHC_574	*Micropterus nigricans*	29.51405	-99.81174	TNHC 55169	TNHC_574
M_nigricans_TNHC_575	*Micropterus nigricans*	29.51405	-99.81174	TNHC 55169	TNHC_575
M_nigricans_TNHC_679	*Micropterus nigricans*	29.61613	-99.74451	TNHC 56829	TNHC_679
M_nigricans_TNHC_1007	*Micropterus nigricans*	28.49660	-96.84360	TNHC 61120	TNHC_1007
M_nigricans_TNHC_570	*Micropterus nigricans*	29.44319	-99.99377	TNHC 55109	TNHC_570
M_nigricans_TNHC_1606	*Micropterus nigricans*	30.13040	-101.57430	TNHC 65057	TNHC_1606
M_nigricans_TNHC_1616	*Micropterus nigricans*	30.13040	-101.57430	TNHC 65057	TNHC_1616
M_nigricans_TNHC_1620	*Micropterus nigricans*	30.13040	-101.57430	TNHC 64934	TNHC_1620
M_nigricans_TNHC_1643	*Micropterus nigricans*	30.44240	-101.72030	TNHC 64928	TNHC_1643
M_nigricans_TNHC_1649	*Micropterus nigricans*	30.45060	-101.73110	TNHC 65071	TNHC_1649
M_nigricans_TNHC_1650	*Micropterus nigricans*	30.45060	-101.73110	TNHC 65071	TNHC_1650
M_nigricans_TNHC_1651	*Micropterus nigricans*	30.45060	-101.73110	TNHC 65071	TNHC_1651
M_nigricans_TNHC_1667	*Micropterus nigricans*	30.46920	-101.80270	TNHC 65115	TNHC_1667
M_nigricans_TNHC_1677	*Micropterus nigricans*	30.46060	-101.82510	TNHC 65172	TNHC_1677
M_nigricans_TNHC_966	*Micropterus nigricans*	30.78860	-101.83540	TNHC 62131	TNHC_966
M_nigricans_TNHC_928	*Micropterus nigricans*	34.84830	-100.06330	TNHC 59293	TNHC_928
M_nigricans_YFTC_38151	*Micropterus nigricans*	38.76609	-77.97687	YPM ICH 033630	YFTC_38151
M_nigricans_X_M_salmoides_YFTC_38184	*Micropterus nigricans*	38.36938	-77.79953	YPM ICH 033643	YFTC_38184
M_nigricans_X_M_salmoides_YFTC_38185	*Micropterus nigricans*	38.36938	-77.79953	YPM ICH 033643	YFTC_38185
M_nigricans_X_M_salmoides_YFTC_38186	*Micropterus nigricans*	38.36938	-77.79953	YPM ICH 033643	YFTC_38186

M_nigricans_X_M_salmoides_YFTC_38189	*Micropterus nigricans*	38.36938	-77.79953	YPM ICH 033643	YFTC_38189
M_nigricans_X_M_salmoides_YFTC_38319	*Micropterus nigricans*	38.01856	-77.28130	YPM ICH 033743	YFTC_38319
M_nigricans_X_M_salmoides_YFTC_38320	*Micropterus nigricans*	38.01856	-77.28130	YPM ICH 033743	YFTC_38320
M_nigricans_X_M_salmoides_YFTC_38353	*Micropterus nigricans*	37.75965	-77.61236	YPM ICH 033580	YFTC_38353
M_nigricans_X_M_salmoides_YFTC_38373	*Micropterus nigricans*	37.75965	-77.61236	YPM ICH 033639	YFTC_38373
M_nigricans_YFTC_38386	*Micropterus nigricans*	44.89468	-73.26783	YPM ICH 033918	YFTC_38386
M_notius_YFTC_31843	*Micropterus notius*	30.14764	-83.96900	YPM ICH 031549	YFTC_31843
M_notius_YFTC_31845	*Micropterus notius*	30.14764	-83.96900	YPM ICH 031549	YFTC_31845
M_notius_YFTC_31846	*Micropterus notius*	30.14764	-83.96900	YPM ICH 031549	YFTC_31846
M_notius_YFTC_31847	*Micropterus notius*	30.14764	-83.96900	YPM ICH 031549	YFTC_31847
M_notius_YFTC_31850	*Micropterus notius*	30.14764	-83.96900	YPM ICH 031549	YFTC_31850
M_notius_YFTC_31851	*Micropterus notius*	30.14764	-83.96900	YPM ICH 031549	YFTC_31851
M_notius_YFTC_31852	*Micropterus notius*	30.14764	-83.96900	YPM ICH 031549	YFTC_31852
M_notius_YFTC_31853	*Micropterus notius*	30.14764	-83.96900	YPM ICH 031549	YFTC_31853
M_notius_YFTC_31854	*Micropterus notius*	30.14764	-83.96900	YPM ICH 031549	YFTC_31854
M_notius_UF_181353	*Micropterus notius*	29.86928	-82.74431	UF 181353	UF_181353
M_notius_UF_170650	*Micropterus notius*	30.20278	-83.96917	UF 170650	UF_170650
M_notius_UF_183662	*Micropterus notius*	30.20278	-83.96917	UF 183662	UF_183662
M_notius_YFTC_31807	*Micropterus notius*	29.86897	-82.74376	YPM ICH 031418	YFTC_31807
M_punctulatus_YFTC_13075	*Micropterus punctulatus*	32.35522	-85.09525	YPM ICH 020718	YFTC_13075
M_punctulatus_YFTC_2811	*Micropterus punctulatus*	34.25087	-96.54928	UT 90.3444	YFTC_2811
M_punctulatus_YFTC_8332	*Micropterus punctulatus*	36.58436	-84.23305	YPM ICH 015481	YFTC_8332
M_punctulatus_YFTC_8333	*Micropterus punctulatus*	36.58436	-84.23305	YPM ICH 015481	YFTC_8333
M_punctulatus_UF_238439	*Micropterus punctulatus*	30.12935	-85.14460	UF 238439	UF_238439

M_punctulatus_YFTC_10677	*Micropterus punctulatus*	34.95698	-92.98479	YPM ICH 018722	YFTC_10677
M_punctulatus_YFTC_10070	*Micropterus punctulatus*	37.25390	-85.50249	YPM ICH 017635	YFTC_10070
M_punctulatus_YFTC_1512	*Micropterus punctulatus*	37.51076	-89.37737	NA	YFTC_1512
M_punctulatus_YFTC_1513	*Micropterus punctulatus*	37.51076	-89.37737	NA	YFTC_1513
M_punctulatus_KSDNR_SPB1	*Micropterus punctulatus*	38.43727	-96.55407	NA	KS_SPB1
M_punctulatus_KSDNR_SPB2	*Micropterus punctulatus*	38.43727	-96.55407	NA	KS_SPB2
M_punctulatus_UF_165437	*Micropterus punctulatus*	37.11223	-85.62584	UF 165437	UF_165437
M_punctulatus_UF_165467	*Micropterus punctulatus*	36.65440	-85.81794	UF 165467	UF_165467
M_punctulatus_YFTC_6303	*Micropterus punctulatus*	36.92693	-87.25648	NA	YFTC_6303
M_punctulatus_YFTC_6304	*Micropterus punctulatus*	36.92693	-87.25648	NA	YFTC_6304
M_punctulatus_YFTC_2864	*Micropterus punctulatus*	34.42689	-95.57836	UT 90.3470	YFTC_2864
M_punctulatus_YFTC_2865	*Micropterus punctulatus*	34.42689	-95.57836	UT 90.3470	YFTC_2865
M_punctulatus_MW_D5	*Micropterus punctulatus*	30.45580	-90.10470	NA	MW_D5
M_punctulatus_MW_D6	*Micropterus punctulatus*	30.45580	-90.10470	NA	MW_D6
M_punctulatus_MW_D7	*Micropterus punctulatus*	30.45580	-90.10470	NA	MW_D7
M_punctulatus_MW_D8	*Micropterus punctulatus*	30.45580	-90.10470	NA	MW_D8
M_punctulatus_MW_E5	*Micropterus punctulatus*	32.70774	-90.09378	NA	MW_E5
M_punctulatus_MW_E6	*Micropterus punctulatus*	32.70774	-90.09378	NA	MW_E6
M_punctulatus_MW_E7	*Micropterus punctulatus*	32.60351	-90.36390	NA	MW_E7
M_punctulatus_MW_E8	*Micropterus punctulatus*	32.60351	-90.36390	NA	MW_E8
M_punctulatus_MMNS_4864	*Micropterus punctulatus*	31.26153	-88.90308	MMNS 67341 (TN-1)	MMNS_4864
M_punctulatus_MMNS_4865	*Micropterus punctulatus*	31.26153	-88.90308	MMNS 67341 (TN-2)	MMNS_4865
M_punctulatus_MMNS_4866	*Micropterus punctulatus*	31.26153	-88.90308	MMNS 67341 (TN-3)	MMNS_4866
M_punctulatus_MMNS_4867	*Micropterus punctulatus*	31.26153	-88.90308	MMNS 67341 (TN-4)	MMNS_4867

M_punctulatus_MMNS_4869	*Micropterus punctulatus*	31.26153	-88.90308	MMNS 67341 (TN-6)	MMNS_4869
M_punctulatus_MMNS_4874	*Micropterus punctulatus*	31.51618	-90.03494	MMNS 67343 (P1)	MMNS_4874
M_punctulatus_MMNS_4878	*Micropterus punctulatus*	31.51618	-90.03494	MMNS 67343 (P5)	MMNS_4878
M_punctulatus_MMNS_4881	*Micropterus punctulatus*	31.51618	-90.03494	MMNS 67343 (P8)	MMNS_4881
M_punctulatus_YFTC_10221	*Micropterus punctulatus*	38.32812	-91.30255	YPM ICH 020815	YFTC_10221
M_punctulatus_TCWC_17554-13.1	*Micropterus punctulatus*	30.40000	-94.26000	TCWC 17554-13.1	TCWC_17554-13.1
M_punctulatus_TCWC_17554-13.2	*Micropterus punctulatus*	30.40000	-94.26000	TCWC 17554-13.2	TCWC_17554-13.2
M_punctulatus_TCWC_17554-13.3	*Micropterus punctulatus*	30.40000	-94.26000	TCWC 17554-13.3	TCWC_17554-13.3
M_punctulatus_AT_TFAR034	*Micropterus punctulatus*	34.31427	-94.93748	NA	AT_TFAR034
M_punctulatus_AT_TFAR043	*Micropterus punctulatus*	34.30878	-94.93570	NA	AT_TFAR043
M_punctulatus_AT_GLVR019	*Micropterus punctulatus*	35.83623	-94.91812	NA	AT_GLVR019
M_punctulatus_AT_GLVR020	*Micropterus punctulatus*	35.83623	-94.91812	NA	AT_GLVR020
M_punctulatus_AT_SPRG079	*Micropterus punctulatus*	36.87100	-94.76551	NA	AT_SPRG079
M_punctulatus_AT_SPRG080	*Micropterus punctulatus*	36.87100	-94.76551	NA	AT_SPRG080
M_punctulatus_YFTC_15663	*Micropterus punctulatus*	30.45532	-90.10522	SELU 6337	YFTC_15663
M_punctulatus_YFTC_15665	*Micropterus punctulatus*	30.45532	-90.10522	SELU 6337	YFTC_15665
M_punctulatus_YFTC_32094	*Micropterus punctulatus*	30.48359	-90.55675	YPM ICH 031573	YFTC_32094
M_punctulatus_YFTC_4945	*Micropterus punctulatus*	31.36477	-93.68563	YPM ICH 021180	YFTC_4945
M_punctulatus_YFTC_4946	*Micropterus punctulatus*	31.36477	-93.68563	YPM ICH 021180	YFTC_4946
M_punctulatus_YFTC_10112	*Micropterus punctulatus*	36.74109	-88.46171	YPM ICH 018181	YFTC_10112
M_punctulatus_UF_167922	*Micropterus punctulatus*	36.59612	-87.05993	UF 167922	UF_167922
M_punctulatus_YFTC_3420	*Micropterus punctulatus*	35.39727	-88.96834	NA	YFTC_3420
M_punctulatus_YFTC_3442	*Micropterus punctulatus*	35.27371	-88.91333	NA	YFTC_3442
M_punctulatus_YFTC_3443	*Micropterus punctulatus*	35.27371	-88.91333	NA	YFTC_3443

M_punctulatus_YFTC_3444	*Micropterus punctulatus*	35.27371	-88.91333	NA	YFTC_3444
M_punctulatus_YFTC_3445	*Micropterus punctulatus*	35.27371	-88.91333	NA	YFTC_3445
M_punctulatus_YFTC_3446	*Micropterus punctulatus*	35.27371	-88.91333	NA	YFTC_3446
M_punctulatus_TNHC_1683	*Micropterus punctulatus*	30.81340	-97.25530	TNHC 65236	TNHC_1683
M_punctulatus_TNHC_1684	*Micropterus punctulatus*	30.81340	-97.25530	TNHC 65236	TNHC_1684
M_punctulatus_TNHC_1711	*Micropterus punctulatus*	31.12461	-97.23254	TNHC 65264	TNHC_1711
M_punctulatus_TNHC_1715	*Micropterus punctulatus*	31.12461	-97.23254	TNHC 65264	TNHC_1715
M_punctulatus_TNHC_596	*Micropterus punctulatus*	31.84447	-97.20150	TNHC 55324	TNHC_596
M_punctulatus_TNHC_159	*Micropterus punctulatus*	30.58280	-99.31970	TNHC 47890	TNHC_159
M_punctulatus_TNHC_303	*Micropterus punctulatus*	30.22257	-97.40945	TNHC 47932	TNHC_303
M_punctulatus_YFTC_2477	*Micropterus punctulatus*	35.18041	-88.20965	UT 90.3331	YFTC_2477
M_salmoides_YFTC_21513	*Micropterus salmoides*	27.67153	-82.35422	YPM ICH 026208	YFTC_21513
M_salmoides_YFTC_21514	*Micropterus salmoides*	27.67153	-82.35422	YPM ICH 026208	YFTC_21514
M_salmoides_YFTC_21449	*Micropterus salmoides*	29.08078	-81.56747	YPM ICH 025176	YFTC_21449
M_salmoides_YFTC_21463	*Micropterus salmoides*	29.08078	-81.56747	YPM ICH 025180	YFTC_21463
M_salmoides_YFTC_21464	*Micropterus salmoides*	29.08078	-81.56747	YPM ICH 025180	YFTC_21464
M_salmoides_YFTC_21465	*Micropterus salmoides*	29.08078	-81.56747	YPM ICH 025180	YFTC_21465
M_salmoides_YFTC_21466	*Micropterus salmoides*	29.08078	-81.56747	YPM ICH 025180	YFTC_21466
M_salmoides_YFTC_31870	*Micropterus salmoides*	30.44071	-84.98630	YPM ICH 031356	YFTC_31870
M_salmoides_YFTC_31871	*Micropterus salmoides*	30.44071	-84.98630	YPM ICH 031356	YFTC_31871
M_salmoides_YFTC_31872	*Micropterus salmoides*	30.44071	-84.98630	YPM ICH 031356	YFTC_31872
M_salmoides_YFTC_31873	*Micropterus salmoides*	30.44071	-84.98630	YPM ICH 031356	YFTC_31873
M_salmoides_YFTC_31874	*Micropterus salmoides*	30.44071	-84.98630	YPM ICH 031356	YFTC_31874
M_salmoides_YFTC_31875	*Micropterus salmoides*	30.44071	-84.98630	YPM ICH 031356	YFTC_31875

M_salmoides_YFTC_31876	*Micropterus salmoides*	30.44071	-84.98630	YPM ICH 031356	YFTC_31876
M_salmoides_YFTC_31877	*Micropterus salmoides*	30.44071	-84.98630	YPM ICH 031356	YFTC_31877
M_salmoides_YFTC_31878	*Micropterus salmoides*	30.44071	-84.98630	YPM ICH 031356	YFTC_31878
M_salmoides_UF_183556	*Micropterus salmoides*	30.15569	-84.21283	UF 183556	UF_183556
M_salmoides_YFTC_31606	*Micropterus salmoides*	26.72374	-81.69197	YPM ICH 031497	YFTC_31606
M_salmoides_YFTC_31607	*Micropterus salmoides*	26.72374	-81.69197	YPM ICH 031497	YFTC_31607
M_salmoides_YFTC_31611	*Micropterus salmoides*	26.72374	-81.69197	YPM ICH 031497	YFTC_31611
M_salmoides_YFTC_31613	*Micropterus salmoides*	26.72374	-81.69197	YPM ICH 031497	YFTC_31613
M_salmoides_YFTC_31614	*Micropterus salmoides*	26.72374	-81.69197	YPM ICH 031497	YFTC_31614
M_salmoides_YFTC_31615	*Micropterus salmoides*	26.72374	-81.69197	YPM ICH 031497	YFTC_31615
M_salmoides_YFTC_31616	*Micropterus salmoides*	26.72374	-81.69197	YPM ICH 031497	YFTC_31616
M_salmoides_YFTC_31617	*Micropterus salmoides*	26.72374	-81.69197	YPM ICH 031497	YFTC_31617
M_salmoides_YFTC_31618	*Micropterus salmoides*	26.72374	-81.69197	YPM ICH 031497	YFTC_31618
M_salmoides_YFTC_31619	*Micropterus salmoides*	26.72374	-81.69197	YPM ICH 031497	YFTC_31619
M_salmoides_YFTC_31620	*Micropterus salmoides*	26.72374	-81.69197	YPM ICH 031497	YFTC_31620
M_salmoides_YFTC_31621	*Micropterus salmoides*	26.72374	-81.69197	YPM ICH 031497	YFTC_31621
M_salmoides_X_M_nigricans_YFTC_31912	*Micropterus salmoides*	30.80182	-85.82888	YPM ICH 031464	YFTC_31912
M_salmoides_X_M_nigricans_YFTC_31923	*Micropterus salmoides*	30.80182	-85.82888	YPM ICH 031671	YFTC_31923
M_salmoides_X_M_nigricans_YFTC_31924	*Micropterus salmoides*	30.80182	-85.82888	YPM ICH 031671	YFTC_31924
M_salmoides_X_M_nigricans_YFTC_31925	*Micropterus salmoides*	30.80182	-85.82888	YPM ICH 031671	YFTC_31925
M_salmoides_X_M_nigricans_YFTC_31929	*Micropterus salmoides*	30.80182	-85.82888	YPM ICH 031671	YFTC_31929
M_salmoides_X_M_nigricans_YFTC_31930	*Micropterus salmoides*	30.80182	-85.82888	YPM ICH 031671	YFTC_31930
M_salmoides_X_M_nigricans_YFTC_31931	*Micropterus salmoides*	30.80182	-85.82888	YPM ICH 031671	YFTC_31931
M_salmoides_X_M_nigricans_YFTC_31932	*Micropterus salmoides*	30.80182	-85.82888	YPM ICH 031671	YFTC_31932

M_salmoides_X_M_nigricans_YFTC_31933	*Micropterus salmoides*	30.80182	-85.82888	YPM ICH 031671	YFTC_31933
M_salmoides_X_M_nigricans_YFTC_31934	*Micropterus salmoides*	30.80182	-85.82888	YPM ICH 031671	YFTC_31934
M_salmoides_X_M_nigricans_YFTC_31935	*Micropterus salmoides*	30.80182	-85.82888	YPM ICH 031671	YFTC_31935
M_salmoides_X_M_nigricans_YFTC_31936	*Micropterus salmoides*	30.80182	-85.82888	YPM ICH 031671	YFTC_31936
M_salmoides_YFTC_31601	*Micropterus salmoides*	27.34407	-82.15678	YPM ICH 031534	YFTC_31601
M_salmoides_YFTC_31602	*Micropterus salmoides*	27.34407	-82.15678	YPM ICH 031534	YFTC_31602
M_salmoides_YFTC_31603	*Micropterus salmoides*	27.34407	-82.15678	YPM ICH 031534	YFTC_31603
M_salmoides_YFTC_31691	*Micropterus salmoides*	29.04922	-82.44736	YPM ICH 031524	YFTC_31691
M_salmoides_YFTC_31692	*Micropterus salmoides*	29.04922	-82.44736	YPM ICH 031524	YFTC_31692
M_salmoides_YFTC_31693	*Micropterus salmoides*	29.04922	-82.44736	YPM ICH 031524	YFTC_31693
M_salmoides_UF_162702	*Micropterus salmoides*	26.21472	-80.28000	UF 162702	UF_162702
M_salmoides_UF_158845	*Micropterus salmoides*	29.86056	-82.22944	UF 158845	UF_158845
M_salmoides_YFTC_31525	*Micropterus salmoides*	31.42717	-81.60641	YPM ICH 031561	YFTC_31525
M_salmoides_YFTC_31526	*Micropterus salmoides*	31.42717	-81.60641	YPM ICH 031561	YFTC_31526
M_salmoides_YFTC_31527	*Micropterus salmoides*	31.42717	-81.60641	YPM ICH 031561	YFTC_31527
M_salmoides_YFTC_31552	*Micropterus salmoides*	31.22039	-81.86717	YPM ICH 031537	YFTC_31552
M_salmoides_YFTC_31553	*Micropterus salmoides*	31.22039	-81.86717	YPM ICH 031537	YFTC_31553
M_salmoides_X_M_nigricans_YFTC_37726	*Micropterus salmoides*	35.57329	-78.97571	YPM ICH 033045	YFTC_37726
M_salmoides_X_M_nigricans_YFTC_37727	*Micropterus salmoides*	35.57329	-78.97571	YPM ICH 033045	YFTC_37727
M_salmoides_X_M_nigricans_YFTC_35853	*Micropterus salmoides*	35.75319	-81.89163	YPM ICH 033437	YFTC_35853
M_salmoides_YFTC_37259	*Micropterus salmoides*	34.30055	-78.55161	YPM ICH 032712	YFTC_37259
M_salmoides_YFTC_37637	*Micropterus salmoides*	36.19096	-76.46647	YPM ICH 032649	YFTC_37637
M_salmoides_YFTC_37638	*Micropterus salmoides*	36.19096	-76.46647	YPM ICH 032649	YFTC_37638
M_salmoides_X_M_nigricans_YFTC_29901	*Micropterus salmoides*	36.38263	-79.98060	YPM ICH 031111	YFTC_29901

M_salmoides_X_M_nigricans_YFTC_35413	*Micropterus salmoides*	35.43880	-79.99930	YPM ICH 033497	YFTC_35413
M_salmoides_YFTC_6197	*Micropterus salmoides*	32.49464	-81.55544	UT 90.3686	YFTC_6197
M_salmoides_YFTC_6198	*Micropterus salmoides*	32.49464	-81.55544	UT 90.3686	YFTC_6198
M_salmoides_SCDNR_MISA00001	*Micropterus salmoides*	32.95786	-80.20058	NA	SCDNR_MISA00001
M_salmoides_SCDNR_MISA00002	*Micropterus salmoides*	32.95786	-80.20058	NA	SCDNR_MISA00002
M_salmoides_SCDNR_MISA00003	*Micropterus salmoides*	32.95786	-80.20058	NA	SCDNR_MISA00003
M_salmoides_SCDNR_MISA00004	*Micropterus salmoides*	32.95786	-80.20058	NA	SCDNR_MISA00004
M_salmoides_SCDNR_MISA00006	*Micropterus salmoides*	32.95786	-80.20058	NA	SCDNR_MISA00006
M_salmoides_SCDNR_MISA00007	*Micropterus salmoides*	33.05735	-79.95840	NA	SCDNR_MISA00007
M_salmoides_SCDNR_MISA00005	*Micropterus salmoides*	32.67926	-80.41730	NA	SCDNR_MISA00005
M_salmoides_X_M_nigricans_YFTC_4947	*Micropterus salmoides*	31.36477	-93.68563	YPM ICH 021177	YFTC_4947
M_salmoides_YFTC_6125	*Micropterus salmoides*	33.27752	-82.29718	UT 90.3712	YFTC_6125
M_salmoides_YFTC_6126	*Micropterus salmoides*	33.27752	-82.29718	UT 90.3712	YFTC_6126
M_salmoides_YFTC_6127	*Micropterus salmoides*	33.27752	-82.29718	UT 90.3712	YFTC_6127
M_salmoides_YFTC_16737	*Micropterus salmoides*	34.25844	-78.52364	YPM ICH 028445	YFTC_16737
M_salmoides_YFTC_16746	*Micropterus salmoides*	34.25844	-78.52364	YPM ICH 028438	YFTC_16746
M_tallapoosae_YFTC_19301	*Micropterus tallapoosae*	33.59300	-85.26390	GMNHTC 8637	YFTC_19301
M_tallapoosae_YFTC_35174	*Micropterus tallapoosae*	33.76287	-85.22267	YPM ICH 033313	YFTC_35174
M_tallapoosae_YFTC_35176	*Micropterus tallapoosae*	33.76287	-85.22267	YPM ICH 033313	YFTC_35176
M_tallapoosae_YFTC_35177	*Micropterus tallapoosae*	33.76287	-85.22267	YPM ICH 033313	YFTC_35177
M_treculii_X_M_punctulatus_YFTC_402	*Micropterus treculii*	29.53572	-97.88082	INHS 40486	YFTC_402
M_treculii_TCWC_17548-01	*Micropterus treculii*	29.97000	-101.15000	TCWC 17548-01	TCWC_17548-01
M_treculii_YFTC_399	*Micropterus treculii*	29.94875	-98.92572	INHS 41173	YFTC_399
M_treculii_YFTC_24670	*Micropterus treculii*	30.47059	-99.78509	YPM ICH 027988	YFTC_24670

M_treculii_TCWC_17549-03	*Micropterus treculii*	30.91886	-99.78382	TCWC 17549-03	TCWC_17549-03
M_treculii_TNHC_1333	*Micropterus treculii*	30.00440	-97.95460	TNHC 63180	TNHC_1333
M_treculii_TNHC_1698	*Micropterus treculii*	30.90180	-96.95830	TNHC 64922	TNHC_1698
M_treculii_TNHC_401	*Micropterus treculii*	30.57820	-99.32330	TNHC 47410	TNHC_401
M_treculii_TNHC_402	*Micropterus treculii*	30.57820	-99.32330	TNHC 47410	TNHC_402
M_treculii_TNHC_571	*Micropterus treculii*	29.72085	-100.03326	TNHC 55105	TNHC_571
M_velox_X_M_dolomieu_AT_BIGSU014	*Micropterus velox*	36.61673	-94.35070	NA	AT_BIGSU014
M_velox_AT_INDI031	*Micropterus velox*	36.79318	-94.24312	NA	AT_INDI031
M_velox_X_M_dolomieu_AT_SMBGR073	*Micropterus velox*	36.55491	-94.49785	NA	AT_SMBGR073
M_velox_X_M_dolomieu_AT_SHOAL011	*Micropterus velox*	36.92026	-94.34571	NA	AT_SHOAL011
M_velox_AT_BFORK013	*Micropterus velox*	35.93656	-94.82767	NA	AT_BFORK013
M_velox_AT_SMBGR016	*Micropterus velox*	36.54624	-94.68764	NA	AT_SMBGR016
M_velox_AT_GRSPB004	*Micropterus velox*	35.84151	-94.77270	NA	AT_GRSPB004
M_velox_AT_NOIS017	*Micropterus velox*	35.84151	-94.77270	NA	AT_NOIS017
M_velox_AT_BFC014	*Micropterus velox*	35.56405	-94.53228	NA	AT_BFC014
M_velox_AT_SPVW013	*Micropterus velox*	36.32432	-94.69931	NA	AT_SPVW013
M_velox_AT_CLPD1	*Micropterus velox*	36.77426	-94.68473	NA	AT_CLPD1
M_warriorensis_YFTC_36235	*Micropterus warriorensis*	33.52186	-87.48497	YPM ICH 032201	YFTC_36235
M_warriorensis_YFTC_32217	*Micropterus warriorensis*	33.91751	-86.81700	YPM ICH 031737	YFTC_32217
M_warriorensis_YFTC_32218	*Micropterus warriorensis*	33.91751	-86.81700	YPM ICH 031737	YFTC_32218
M_warriorensis_YFTC_32225	*Micropterus warriorensis*	33.91751	-86.81700	YPM ICH 031737	YFTC_32225
M_warriorensis_YFTC_37096	*Micropterus warriorensis*	34.28514	-87.39899	YPM ICH 031861	YFTC_37096
M_warriorensis_YFTC_37097	*Micropterus warriorensis*	34.28514	-87.39899	YPM ICH 031861	YFTC_37097

Supplementary Table S2. Pairwise *Fst* (above diagonal) and Nei's standard genetic distance (below diagonal) values of combined ddRAD loci for the 19 delimited species of *Micropterus* in the present study. Species abbreviations: Malt, *Micropterus* cf. *coosae* Altamaha; Mcah, *Micropterus cahabae*; Mcat, *Micropterus cataractae*; Mcho, *Micropterus* cf. *punctulatus*; Mcht, *Micropterus chattahoochae*; Mcoo, *Micropterus coosae*; Mdol, *Micropterus dolomieu*; Mlit, *Micropterus* cf. *dolomieu* Little; Moua, *Micropterus* cf. *dolomieu* Ouachita; Mvel, *Micropterus velox*; Msal, *Micropterus salmoides*; Mhen, *Micropterus henshalli*; Mnot, *Micropterus notius*; Mpun, *Micropterus punctulatus*; Mnig, *Micropterus nigricans*; Mbrt, *Micropterus* cf. *coosae* Bartram's; Mtal, *Micropterus tallapoosae*; Mtre, *Micropterus treculii*; Mwar, *Micropterus warriorensis*.

Species	Malt	Mcah	Mcat	Mcho	Mcht	Mcoo	Mdol	Mlit	Moua	Mvel	Msal	Mhen	Mnot	Mpun	Mnig	Mbrt	Mtal	Mtre	Mwar
Malt		0.64	0.90	0.80	0.41	0.62	0.78	0.85	0.87	0.81	0.82	0.77	0.96	0.81	0.72	0.47	0.70	0.77	0.77
Mcah	0.03		0.84	0.68	0.44	0.24	0.73	0.76	0.79	0.76	0.80	0.64	0.92	0.75	0.69	0.61	0.34	0.69	0.61
Mcat	0.08	0.07		0.87	0.80	0.83	0.81	0.88	0.89	0.86	0.83	0.85	0.95	0.85	0.74	0.86	0.87	0.83	0.88
Mcho	0.05	0.04	0.07		0.64	0.68	0.74	0.80	0.83	0.78	0.81	0.55	0.94	0.64	0.70	0.76	0.73	0.58	0.76
Mcht	0.02	0.02	0.06	0.04		0.45	0.71	0.72	0.75	0.72	0.79	0.62	0.91	0.72	0.68	0.40	0.44	0.66	0.59
Mcoo	0.03	0.01	0.07	0.04	0.02		0.73	0.76	0.78	0.75	0.80	0.64	0.91	0.75	0.69	0.59	0.32	0.70	0.59
Mdol	0.07	0.06	0.07	0.06	0.05	0.06		0.38	0.36	0.49	0.81	0.73	0.88	0.75	0.73	0.77	0.74	0.74	0.76
Mlit	0.07	0.06	0.08	0.06	0.06	0.06	0.01		0.44	0.57	0.82	0.78	0.94	0.79	0.72	0.82	0.80	0.76	0.82
Moua	0.07	0.06	0.08	0.06	0.06	0.06	0.01	0.01		0.60	0.82	0.80	0.95	0.81	0.73	0.83	0.82	0.78	0.84
Mvel	0.07	0.06	0.08	0.06	0.06	0.06	0.02	0.02	0.02		0.82	0.77	0.92	0.80	0.73	0.80	0.77	0.77	0.79
Msal	0.09	0.08	0.09	0.08	0.08	0.08	0.09	0.09	0.09	0.09		0.80	0.87	0.82	0.41	0.82	0.80	0.81	0.81
Mhen	0.05	0.03	0.07	0.02	0.03	0.03	0.05	0.06	0.06	0.06	0.08		0.92	0.60	0.70	0.74	0.68	0.55	0.71
Mnot	0.12	0.11	0.11	0.11	0.11	0.11	0.11	0.11	0.12	0.12	0.12	0.11		0.91	0.80	0.94	0.94	0.90	0.94
Mpun	0.06	0.04	0.07	0.03	0.04	0.05	0.05	0.05	0.06	0.06	0.09	0.02	0.11		0.74	0.80	0.77	0.51	0.78
Mnig	0.09	0.07	0.08	0.08	0.07	0.07	0.08	0.08	0.08	0.09	0.02	0.07	0.11	0.08		0.72	0.69	0.72	0.71

Mbrt	0.01	0.03	0.07	0.05	0.02	0.03	0.06	0.07	0.07	0.07	0.09	0.05	0.12	0.06	0.08		0.65	0.75	0.72
Mtal	0.03	0.01	0.07	0.04	0.02	0.01	0.06	0.06	0.06	0.07	0.08	0.04	0.11	0.05	0.08	0.03		0.72	0.65
Mtre	0.06	0.04	0.07	0.02	0.04	0.05	0.05	0.06	0.06	0.06	0.09	0.02	0.11	0.02	0.08	0.05	0.05		0.74
Mwar	0.04	0.03	0.07	0.05	0.03	0.03	0.06	0.06	0.07	0.07	0.09	0.04	0.11	0.05	0.08	0.04	0.03	0.05	

Supplementary Table S3. Models tested in the Fastsimcoal2 analysis for the Spotted Bass complex. Parameter estimates of the highest estimated log-likelihood (MaxEstLhood) for each model are shown. Best-fit model for each population is highlighted. Species, populations, and model parameter abbreviations correspond to those shown in Supplementary Fig. S4. Species or population names and ANCSIZE are population size parameters. Timing parameters for divergence (TDIV), migration (TMIG and Tstop), and human-mediated introduction (TINT) are in the number of generations, assuming a generation time of three years. The mean and 95% confidence interval of each parameter from 100 parametric bootstrap replications is shown for models highlighted in bold.

Topology 'Texas1' (MaxObsLhood: -13780.4; AIC: 30372)											
ANCSIZE	Mpun	MpunSN	MpunBZ	Mtre	TDIV_sn_pu	TDIV_bz_tr	MaxEstLhood				
1453139	625484	1147728	1855270	385447	357440	271951	-15179				
Topology 'Texas2' (AIC: 30506.9)											
ANCSIZE	Mpun	MpunSN	MpunBZ	Mtre	TDIV_sn_tr	TDIV_bz_tr	MaxEstLhood				
1355873	700680	1071720	2085302	339545	398475	233690	-15246.5				
Gene-flow 'Texas1' (MaxObsLhood: -13780.4; AIC: 29362)											
ANCSIZE	Mpun	MpunSN	MpunBZ	Mtre	s_bz	TDIV_sn_pu	TDIV_bz_tr	TMIG	MaxEstLhood		
801700	443023	941938	145750	294386	0.26652	250136	99849	7066	-14672		
**Gene-flow 'Texas2' (AIC: 29354.8)**											
	ANCSIZE	Mpun	MpunSN	MpunBZ	Mtre	s_bz	b_sn	TDIV_sn_pu	TDIV_bz_tr	TMIG	MaxEstLhood
	788703	439147	871750	171754	305259	0.261683	6.48E-05	234564	94639	8218	-14667.4
Mean	701290	436100	836920	180967	306350	0.26	3.00E-04	235112	97000	8805	

Lower 95%	596917	399443	722075	142493	285008	0.24	9.99E-08	218562	83425	5920
Upper 95%	788703	472991	936467	236454	329756	0.28	2.18E-03	251693	114666	12628
Gene-flow 'Texas3' (AIC: 29431.4)										
ANCSIZE	Mpun	MpunSN	MpunBZ	Mtre	s_bz	b_sn	TDIV_sn_pu	TDIV_bz_tr	Tstop	MaxEstLhood
811535	465340	748718	316885	305963	3.79E-06	8.94E-08	229548	95000	2935	-14705.7
Gene-flow 'Texas4' (AIC: 29391.3)										
ANCSIZE	Mpun	MpunSN	MpunBZ	Mtre	TINT	s_bz	b_sn	TDIV_sn_pu	TDIV_bz_tr	MaxEstLhood
837343	460657	1008734	167522	309978	132	0.244617	1.70E-04	242837	99935	-14685.7
Gene-flow 'Texas5' (AIC: 29361.4)										
ANCSIZE	Mpun	MpunSN	MpunBZ	Mtre	s_bz	t_sn	TDIV_sn_pu	TDIV_bz_tr	TMIG	MaxEstLhood
713487	452065	997821	151790	322456	0.256701	0.002489	245075	95014	6256	-14670.7
Gene-flow 'Texas6' (AIC: 29391.7)										
ANCSIZE	Mpun	MpunSN	MpunBZ	Mtre	TINT	s_bz	t_sn	TDIV_sn_pu	TDIV_bz_tr	MaxEstLhood
746385	440670	902787	140795	310317	23	0.25171	0.002231	230431	93934	-14685.8
Gene-flow 'Texas7' (AIC: 29390.9)										
ANCSIZE	Mpun	MpunSN	MpunBZ	Mtre	TINT	s_bz	TDIV_sn_pu	TDIV_bz_tr	MaxEstLhood	
804304	444411	948533	219481	316548	7	0.25738	254684	126298	-14686.4	
Topology 'Spotted_SVDQuartets' (MaxObsLhood: -12386.7; AIC: 26495.8)										
ANCSIZE	Mcho	Mhen	Mpun	Mtre	MaxEstLhood					
1348557	549348	582384	536315	319320	-13242.9					
Topology 'Spotted_IQTREE' (AIC: 26574)										
ANCSIZE	Mcho	Mhen	Mpun	Mtre	TDIV_he_pu	TDIV_tr_pu	MaxEstLhood			
1359828	656985	588414	495859	315560	632941	469845	-13280			
Topology 'Spotted_Gulf1' (MaxObsLhood: -86502.8; AIC: 203012)										
ANCSIZE	Mcho	Mhen	Mpun	Mtre	MpunPP	MpunPS	TDIV_pp_he	TDIV_ps_pp	MaxEstLhood	
1424310	544250	968880	913363	561728	1051035	1010963	559088	244351	-101497	
Topology 'Spotted_Gulf2' (AIC: 201876.9)										
ANCSIZE	Mcho	Mhen	Mpun	Mtre	MpunPP	MpunPS	TDIV_pp_pu	TDIV_ps_pp	MaxEstLhood	
1427227	564379	1208711	835425	511866	958988	949672	395281	232703	-100929	
Topology 'Spotted_Gulf3' (AIC: 202655.4)										
ANCSIZE	Mcho	Mhen	Mpun	Mtre	MpunPP	MpunPS	TDIV_ps_pu	TDIV_pp_pu	MaxEstLhood	
1470645	531059	1249799	896647	503156	979534	1168994	386263	343188	-101319	

Topology 'Spotted_Gulf4' (AIC: 202287.8)												
ANCSIZE	Mcho	Mhen	Mpun	Mtre	MpunPP	MpunPS	TDIV_ps_pu	TDIV_pp_pu	MaxEstLhood			
1547620	571979	1395879	845927	491190	1122078	1292945	501755	428341	-101135			
Topology 'Spotted_Gulf5' (AIC: 205704.1)												
ANCSIZE	Mcho	Mhen	Mpun	Mtre	MpunPP	MpunPS	TDIV_ps_he	TDIV_pp_he	MaxEstLhood			
1499295	569147	849729	1001140	561264	953517	1223066	479814	421566	-102843			
Gene-flow 'Spotted_Gulf1' (MaxObsLhood: -86502.8; AIC: 205956.9)												
ANCSIZE	Mcho	Mhen	Mpun	Mtre	MpunPP	MpunPS	h_ps	h_pp	TDIV_pp_pu	TDIV_ps_pp	TMIG_h_ps	TMIG_h_pp
1444497	475343	849527	921873	569102	700010	792420	2.12E-07	2.31E-04	398704	353724	314566	334310
MaxEstLhood												
-102965												
**Gene-flow 'Spotted_Gulf2' (AIC: 204465.8)**												
	ANCSIZE	Mcho	Mhen	Mpun	Mtre	MpunPP	MpunPS	h_pp	TDIV_pp_pu	TDIV_ps_pp	TMIG_h_pp	MaxEstLhood
	1219452	535868	987258	951864	549556	679243	778276	2.84E-04	395539	193259	350024	-102222
Mean	893078	514823	964720	934269	530092	654994	747491	1.88E-03	390347	188235	340172	
Lower 95%	731493	457831	84989	839679	467582	549457	600529	2.44E-07	348934	159632	296833	
Upper 95%	1086056	544822	1012889	986221	549556	679243	778276	1.61E-02	399494	197304	350024	
Gene-flow 'Spotted_Gulf3' (AIC: 206273.5)												
ANCSIZE	Mcho	Mhen	Mpun	Mtre	MpunPP	MpunPS	h_ps	h_pp	TDIV_pp_pu	TDIV_ps_pp	TMIG_all	MaxEstLhood
1261108	496572	852503	1001072	476782	562035	719469	2.90E-07	1.43E-08	399281	353866	313679	-103125
Gene-flow 'Spotted_Gulf4' (AIC: 206468.8)												
ANCSIZE	Mcho	Mhen	Mpun	Mtre	MpunPP	MpunPS	h_ps	TDIV_pp_pu	TDIV_ps_pp	TMIG	MaxEstLhood	
1367710	545788	1106548	1013642	491120	930091	678935	4.70E-08	396569	350716	310918	-103223	
Gene-flow 'Spotted_Gulf5' (AIC: 217858.7)												
ANCSIZE	Mcho	Mhen	Mpun	Mtre	MpunPP	MpunPS	TINT	h_ps	TDIV_pp_pu	TDIV_ps_pp	MaxEstLhood	
1023494	545177	1561774	884752	500499	728827	1021	48	4.50E-07	317232	184253	-108918	
Gene-flow 'Spotted_Gulf6' (AIC: 226553.7)												
ANCSIZE	Mcho	Mhen	Mpun	Mtre	MpunPP	MpunPS	TINT	h_ps	h_pp	TDIV_pp_pu	TDIV_ps_pp	MaxEstLhood
687106	491289	1644752	822118	581868	161934	1165	49	5.40E-06	2.52E-05	220487	72464	-113265
Topology-geneflow 'Gulf-Texas1' (MaxObsLhood: -11573.4; AIC: 30115.9)												
ANCSIZE	Mhen	Mpun	MpunPP	MpunSN	MpunBZ	Mtre	TDIV_bz_tr	b_sn	s_bz	h_pp	TDIV_pp_pu	TDIV_sn_pu
480055	584491	1016812	535016	588007	188070	272427	118657	3.02E-06	7.50E-07	2.08E-07	397440	303501

TMIG_s_bz	TMIG_h_pp	MaxEstLhood										
67956	341722	-15042.9										
Topology-geneflow 'Gulf-Texas2' (AIC: 30187.8)												
ANCSIZE	Mhen	Mpun	MpunPP	MpunSN	MpunBZ	Mtre	TDIV_bz_tr	b_sn	s_bz	h_pp	TDIV_sn_pu	TDIV_pp_pu
600689	561378	868282	722828	1092270	109820	295603	97375	2.65E-06	9.99E-07	6.76E-08	392460	337876
TMIG_s_bz	TMIG_h_pp	MaxEstLhood										
48783	300827	-15078.9										
